# Industry return lead-lag relationships between the US and other major countries

**DOI:** 10.1186/s40854-022-00439-1

**Published:** 2023-01-17

**Authors:** Ana Monteiro, Nuno Silva, Helder Sebastião

**Affiliations:** CeBER, Faculty of Economics, Univ Coimbra, Av Dias da Silva 165, 3004-512 Coimbra, Portugal

**Keywords:** International diversification, Industry equity indexes, Granger causality, Causality in distribution, C12, G14, G17

## Abstract

In this study, we analyze the lead-lag relationships between the US industry index and those of six other major countries from January 1973 to May 2021. We identify the leading role played by the US internationally by showing that the weekly returns of US industries, especially the US basic materials and energy industries, significantly Granger cause the returns of most other countries’ industries, suggesting that non-US industries react with some delay to new information. This delayed reaction is even more noticeable during periods of recession in the US when cross-country correlations are higher. This implies that the ability of the lagged returns of US industries to predict industries’ returns from other countries is even more pronounced when the US experienced an economic recession. A similar asymmetric relationship is found between the volatility of US industries and that of industries in other markets. The analysis of causality in the distribution of returns and volatility shows that causality runs mainly from the US to other countries, particularly in the presence of extreme negative shocks. Finally, we demonstrate that our predictions are valuable to real-world investors. Long-short strategies generate sizable and statistically significant alphas, and a constant relative risk-averse investor obtains certainty equivalent returns well above the risk-free rate.

## Introduction

In a complete and frictionless market, conventional asset pricing theory assumes that information dissemination across related markets occurs immediately. In a frictionless market with rational expectations, a shock in one asset can be recognized rapidly by investors in other related assets. Consequently, equity prices promptly and fully adjust to information shocks. However, there is compelling empirical evidence that investors face limitations in processing information and non-trivial market frictions. Therefore, contrary to the assumption in conventional theory, information does not spread across markets, (see, for instance, Shiller 2000; Hong et al. 2007). The enormous amount of information decision-makers must process each day is not specific to the financial sector but omnipresent in all economic activities (see, for instance, Kou et al. 2022, who tackle the problem of energy transition in the transport sector). Although the rapid development of information technologies and their widespread adoption by financial firms (e.g., Kou et al. 2021) increase their capacity to gather and process information, it does not solve the problem, as the volume of new information continues to grow and the human ability to interpret it is finite. In a more realistic framework, equity prices may adjust to new information after a delay. For instance, industry-specialized investors may fail to fully assimilate new information from shocks in other industries and global markets initially. Hence, at the industry level, this implies the existence of significant lead-lag relationships between national industry indices and, most notably, between international industries, leading to national and international industry return predictability.

This study analyzes the short-run interdependence between industry returns and volatility in an international context. Previous research has mainly focused on firm-level industry information flows and international global markets (see, for instance, Rapach et al. 2013; Bollerslev et al. 2013, 2014). However, as argued by Hou (2007), market segmentation industry information gradually spreads out over related industries; thus, the returns of an industry can be predicted by the returns of related industries.

The motivation for our study is the desire to contribute to the lead-lag literature. First, to the best of our knowledge, this is the first study to directly address the lead-lag relationship between US industries and other international countries’ industries and examine the economic magnitude of these relationships. Despite the perceived importance of the international linkage between US and non-US returns (see, for instance, Griffin et al. 2011; Rapach et al. 2013; Nyberg and Pönkä 2016), few studies have analyzed or attempted to explain how this causal relationship works and in what direction. Furthermore, there is little understanding of the distinctions in the informational context across countries. This is even more evident at an industrial level. To complete our analysis, we provide fresh insights into the economic impacts of the documented causal relationship. Second, we present a more complete study of the linkages between the international industries of the largest economies in the world (causality and feedback are studied in the mean and the volatility during expansion and recession periods and across quantiles). Third, we use up-to-date data, from January 1, 1973, until May 17, 2021, covering several important events, such as the introduction of the Euro, the subprime mortgage crisis, the European sovereign debt crisis, the US-China trade tension, Brexit, and the outbreak and spread of the COVID-19 pandemic.

Our study refines the work of Rapach et al. (2013) and Ji et al. (2020) on stock market dependence among the G7 countries, the group of seven of the world's most advanced economies whose stock markets should, at least theoretically, show significant co-movement. Over the past 40 years, the importance of the domestic stock market in many industrialized economies has grown sharply while the degree of co-movement among international equity markets seems to have increased. As a result, national economies are more frequently affected by disturbances originating in foreign stock markets and these tend to have far-reaching consequences. Experts argue that financial integration has been spurred by improved electronic communications, the worldwide liberalization of capital controls and financial innovation, and growing political and economic integration.

Lead-lags may arise from information frictions (Aye et al. 2017), the differential response of some stocks to newly released information (Lo and MacKinlay 1990b), or asymmetry in trade and liquidity of assets and markets. Additionally, they may be driven by a behavioral trend-chasing strategy whereby lead-lag effects are a function of the degree to which investors are familiar with the stocks in question (Haque 2011), different paces of adjustment to the various phases of the business cycle (Kanas and Kouretas 2005), different reactions of assets to industry factors (Hou 2007), stocks having time-varying and different sensitivities to common fundamental risk factors (Conrad and Kaul 1988), market microstructure frictions (Boudoukh et al. 1994a), different monetary and fiscal policies (Caporale et al. 2016), or behavioral factors such as irrationality, herding, and gaming behavior (Li et al. 2022).

The study of lead-lags between assets and markets is a hot topic in financial research, and the information provided by international lead-lag relationships is important for investment and policy decision-making. (Asafo-Adjei et al. 2021). The identification of lead-lag network structures and stock characteristics could help practitioners, such as investors and fund managers, identify the information that matters (Fan et al. 2022) and may allow yield enhancement, possibly even arbitrage, through trading futures in some markets (Copeland and Copeland 1998). Furthermore, according to the “wake-up calls” theory, a financial crisis in a given country is a call for investors in another country to acquire information about the risk of exposure to a macro shock in the latter country (Forbes 2012; Ahnert and Bertsch 2022).

It should be noted that in this study, we focus only on the short-run interdependencies. Several studies have analyzed the long-run interdependencies between returns based on the assumption that investors' behavior results in long-run joint stochastic trends between stock markets, which may be captured by cointegration tools. Most notably, Kanas and Kouretas (2005) showed an improvement in the forecasting ability of cointegration between the lagged price of large-firm portfolios and the current price of small-firm portfolios in the UK equity market. Troster et al. (2021) found that the long-term common factor (equilibrium error) between industry portfolios and market cumulative returns has strong predictive power for monthly excess industry portfolio returns in the US. Hence, considering long-run relationships may improve the forecasting ability of international industry returns.

The US plays a key role at the international level. According to the World Bank (2021), the US is the world’s largest national economy, accounting for almost a quarter of the global gross domestic product (GDP), is the most important export destination of most countries worldwide, and represents over one-third of the global stock market capitalization. Because of its size and interconnectivity, events in the US economy are likely to have a global impact. Thus, our main research hypothesis is that lagged US industry returns may help predict the returns of industries in other countries. This is the basic hypothesis of Copeland and Copeland (1998), Rapach et al. (2013), Aye et al. (2017), and Ji et al. (2020). However, this does not mean that feedback from other countries’ industries does not exist (Copeland and Copeland 1998). For instance, Junior et al. (2021) argued that BRIC economies—Brazil, Russia, India, and China—have become increasingly important to the global investment community in recent years due to expectations of growing dominance in the international arena and significant shifts in capital flows into their markets. These can produce good returns during times of stress and may provide a safe haven for returns during times of uncertainty, especially during the COVID-19 pandemic. Emerging economies are also viewed as building blocks for innovation, especially in large developing countries such as China (Asafo-Adjei et al. 2021). Dutta (2018) confirms this statement by finding bidirectional causality between the Chicago Board Options Exchange Volatility Index (VIX) and the implied volatility of the Chinese stock market. Another example of international linkage is provided by Salisu et al. (2022), who studied the linkage between Canada and the US, highlighting that the two economies have strong financial and trading links and that Canada is a potential diversifier for European Union (EU) countries.

We show that the weekly lagged returns of US industries have a strong and significant causal relationship with most other countries considered in the study, whereas the lagged returns of other countries have limited ability to predict US returns at the industry level. Notably, we highlight that the lagged returns of the US basic materials and energy industries have strong and significant predictive power and causality to industries in other countries. This finding is highly plausible because firms in other industries rely heavily on commodities and fuels, and hence the lagged returns of those industries, which are placed earlier in the production chain, should impact the returns of industries positioned later in the production chain.

The leading role of the US is even more pronounced during recession periods when cross-country correlations are stronger. This implies that the ability of US lagged returns to predict the current returns of other countries is much greater when the US experienced a recession in the previous week. The results also suggest that past values of US industries’ volatilities contain information that helps predict the volatility of other countries. We also analyze Granger causality in the distribution of returns and volatilities at the industry level. Our results suggest that other countries did not incorporate shocks affecting US industries in a timely manner, meaning that countries react with a delay to news from the US. Finally, we show that a real-world investor can obtain significant gains using these forecasts.

The remainder of this paper is organized into five sections. Section Literature review presents a brief review of the relevant literature. Section Data description and preliminary analysis presents the data and provides descriptive statistics. Section Methodology outlines the basic theoretical concepts and presents test specifications. Section Empirical Results presents the main results, and Sect. Conclusion concludes the study.

## Literature review

Many academics and practitioners have analyzed the complexity of the relationship between asset prices. For instance, Lo and MacKinlay (1990a) wrote one of the most influential and earliest works in the lead-lag literature in which they showed that the returns of large stocks led to the returns of small stocks in the US from 1962 to 1987. In the nineties, many studies analyzed the lead-lag relationship between various asset prices, industries, and markets (see, e.g., Roll 1992; Arshanapalli and Doukas 1993; Brennan et al. 1993; Boudoukh et al. 1994b; Jegadeesh and Titman 1995; Copeland and Copeland 1998; Moskowitz and Grinblatt 1999). Copeland and Copeland (1998) found that the US had a statistically significant one-day lead over markets in Europe and Asia in the early nineties but no significant lead from other markets. However, this lead did not extend beyond one day. The authors also found that internationalized industries (e.g., airlines) were significantly more sensitive to leads than local ones (e.g., casinos). Moskowitz and Grinblatt (1999) showed that cross-sectional industry momentum accounts for the cross-sectional momentum in individual firm returns, reinforcing the idea that industries have important interconnections with each other.

In the 2000s, many other studies analyzed lead-lag relationships between various industries, between markets (Caporale et al. 2016), or even between an industry (financial sector) and economic growth (Asafo-Adjei et al. 2021). However, they mainly focused on firm-level information flows in the US market. Hou (2007) studied the transmission of information between large and small firms and identified a lead-lag effect between the stock returns of these firms in the US between July 1963 and December 2001. According to the author, this slow information transmission could result from many sources, including incomplete markets, limited stock market participation, asymmetric information, noise trading, limited investor attention, transaction costs, short sale restrictions, legal constraints imposed on institutional investors, and other market frictions. Hong et al. (2007) investigated the transmission of information between US industries and the overall market from January 1946 to December 2002 and concluded that the US stock market reacted to a delay in the information contained in the industry returns about its fundamentals. Consequently, industry returns that incorporate information on macroeconomic fundamentals tend to guide the aggregate market. Hence, a substantial number of US industries, such as retail services, commercial real estate, metals, and petroleum, could anticipate stock market performance for up to two months. Camilleri et al. (2019) discovered a different pattern in Belgium, France, Germany, and the Netherlands. On the one hand, they found contemporaneous and lead-lag relationships between stock prices and the selected variables; however, stock prices significantly lead to inflation across all countries. In addition, stock prices lead to industrial production. Berben and Jansen (2005) present exceptions to this pattern using industry data. The smooth-transition correlation generalized autoregressive conditional heteroskedasticity (STC-GARCH) model applied to weekly data at the industry level from Germany, Japan, the UK, and the US in the period 1980–2000 showed that correlations among the German, UK, and US stock markets have doubled, whereas Japanese correlations have remained the same.

Menzly and Ozbas (2010) found economic links between specific firms and industries that contributed significantly to cross-firm and cross-industry return predictability. In line with Hong et al. (2007), the authors interpreted their findings as evidence of delayed information transmission across economically connected firms and industries. According to Rapach et al. (2015), an industry has an economic link to another if its returns can be predicted by the lagged returns of the other industry. This suggests that shocks in the technology industry, for example, might impact returns in the manufacturing industry, even if these industries are not directly involved with each other. Industries can also be indirectly connected along the production chain, resulting in valuable economic connections that extend beyond the direct customer–supplier link. The authors argue that complex industry interdependencies increase the potential for delayed responses to information and produce cross-industry return predictability.

Beine et al. (2010) used the daily data of 17 developed countries from 1974 to 2006 and combined a quantile regression approach with a subsequent dynamic panel data analysis. The results showed that macroeconomic variables asymmetrically impact stock market co-movement across return distributions. Financial liberalization significantly increased left tail co-movement, whereas trade integration significantly increased co-movement across all quantiles.

Several studies report cross-industry linkages. Rapach et al. (2019) showed that the lagged returns of the financial and commodities industries can be used to forecast in most industries. Jacobsen et al. (2019) demonstrated that industrial metal returns lead the stock market, even after adjusting for other widely used predictors. Additionally, the authors demonstrated that there was a direct relationship between the stock market and past industrial metal returns during recessions and an inverse relationship during expansions. Khalfaoui et al. (2021) analyzed the lead-lag relationships between oil and several metal prices and concluded that gold and platinum were strongly influenced by oil prices, especially during periods of turmoil in global markets. Furthermore, Jiang and Yoon (2020) verified a lead-lag linkage, implying that oil prices led six countries’ stock market indices (China, India, Japan, Saudi Arabia, Russia, and Canada) for periods of 27–30 weeks.

More broadly, Lee et al. (2019) studied the impact of technological proximity on the lead-lag relationship between stock returns and showed that businesses with a positive peer group return in the previous month outperformed those with a negative return. Parsons et al. (2020) documented lead-lag effects on returns between cohead-quartered firms in different industries and uncovered the existence of geographic lead-lags that imply a risk-adjusted return of 5–6% annually, which is half the value observed for industry lead-lag effects. Whereas industry lead-lag effects were stronger among small, thinly traded stocks with low analyst coverage, geographic lead-lags were unrelated to these proxies for investor scrutiny. More recently, Zeng and Mills (2021) reported that economic links, such as customer–supplier relationships and peer effects, only accounted for a small share of the frequently observed cross-firm lead-lag relations. Instead, most cross-firm lead-lag ties are driven by several classical factors.

Ji et al. (2020) analyzed the risk spillover effect between the US stock market and the remaining G7 stock markets by measuring the conditional value-at-risk (CoVaR) using time-varying copula models with Markov switching and data covering a period of more than 100 years. The main results suggest that the dependence structure varies with time and has distinct high- and low-dependence regimes. Their findings validate the existence of risk spillovers between the US stock market and other countries. Abnormal spikes in dynamic CoVaR were induced by well-known historical economic shocks; the value of upside risk spillover is significantly larger than the downside risk spillover, and, surprisingly, the magnitudes of risk spillover from the remaining G7 countries to the US are significantly larger than those from the US, except for Japan, which is the only country with only inflows of Granger causality.

Asafo-Adjei et al. (2021) studied this nexus for Brazil, Russia, India, China, and South Africa (BRICS), showing that the financial sector is the first mover in the long term, except in South Africa. Countries with less developed financial systems have a higher likelihood of experiencing market failure issues due to incomplete competition and information asymmetry. Junior et al. (2021), using daily data from December 11, 2012, to May 28, 2021, performed a bi-wavelet analysis between the BRIC index and its constituents throughout the time–frequency domain. Their findings indicated that the BRIC index was the first variable to respond to shocks, and the co-movements between the BRIC index and its constituents were positive and significant.

Fan et al. (2022) investigated the lead-lag effect from a complex network perspective using data from January 1, 2018, to December 31, 2018, on 218 stocks of the Shanghai Shenzhen CSI 300 index in the Chinese stock market. They detected a lead-lag effect between individual stocks, which was explained by several driving factors such as market capitalization, trading volume, and financial performance.

Although some studies have analyzed lead-lag relationships across industries, they mainly focus on the US market, which is not surprising given that the US is the world's largest stock market. Most of these studies point out that, even for a highly liquid market such as the US, the existence of lead-lag relationships can be interpreted as evidence of information frictions resulting from limited investor attention and limited information-processing capabilities.

An exception to this trend is Rapach et al. (2013), who also reported strong predictability of lagged US monthly index returns over international markets from 1980 to 2010. Additionally, since the US is a large trading partner in many countries and has the world's largest stock market, investors are likely to focus more on the US. As a result, information on US macroeconomic fundamentals is relevant to foreign stock markets. Rapach et al. (2013) also reported that Swedish returns show in-sample predictive power for other foreign returns. This can be justified by the high institutional ownership of Sweden and the fact that institutional investors are more able to collect and process information, which contributes to higher pricing efficiency in the Swedish market.

Wen et al. (2015) also studied international markets and demonstrated that US stock returns could predict South African returns from 1973 to 2014. Cambón and Vaduva (2017) showed that Spanish industries, which provided valuable and important economic information, drove neither equity markets nor economic activity. The hypothesis presented by Hong et al. (2007) was not supported in the case of Spain, where company characteristics, especially size, may be more relevant in understanding lead-lag patterns. Sarwar and Khan (2017) studied the impact of US stock market uncertainty (proxied by the VIX) on Latin American and aggregate emerging markets before, during, and after the 2008 financial crisis. The authors found that increases in the VIX led to significant immediate and delayed declines in emerging market returns in all periods. Tse (2018) examined the lead-lag relationships among 11 industrialized countries using international futures prices and concluded that futures markets were more contemporaneously correlated in market downturns, whereas lead-lag relationships were more significant in market upturns. These findings suggest that investors react more quickly to negative news than positive news. Investors in other countries sell their domestic stocks in the same month when one national stock market falls. In contrast, investors are less likely to buy stocks from other countries when one market performs well.

Understanding whether one company or industry leads another has important implications for investment planning. Croce et al. (2019) showed that firms in leading industries (i.e., industries whose cash flows contain information relevant to future aggregate growth) pay a 4% higher average annualized return than firms in lagging industries. Rapach et al. (2019) reported that a long investment strategy in highly forecastable industries and a short position in the lowest forecastable industries would generate an annualized alpha of at least 8%.

In summary, the extant literature has primarily focused on firm-level returns, intra-industry information flow, and equities and markets. However, industry and firm information spread gradually over related firms and industries. Thus, related industries can predict the returns of an industry (Hou 2007). In addition, studies have primarily focused on the US market rather than on a global scope. However, we know that international markets are now, more than ever, highly connected due to globalization, technological advances, and increasing market integration.

## Data description and preliminary analysis

The data consist of daily closing values of total return equity indexes, in US dollars,^1^ of 11 industries for seven countries, from January 1, 1973, to May 17, 2021, which were retrieved from the Thomson Reuters DataStream database. The period covers the introduction of the Euro, the subprime mortgage crisis, the European sovereign debt crisis, the US-China trade tension, Brexit, and the outbreak and spread of the COVID-19 pandemic. These industries, corresponding to the Level 1 industry classification benchmark (ICB), are basic materials (BM), consumer discretionary (CD), consumer staples (CS), energy (EN), financials (FI), healthcare (HC), industrials (IN), real estate (RE), technology (TEC), telecommunications (TEL), and utilities (UT). We selected the top seven countries according to the Morgan Stanley Capital International All Country World Index (MSCI ACWI): Canada, France, Germany, Japan, China, the UK, and the US. All these countries are considered developed countries and are members of the G7 group, except China. Although China is still considered a developing country, it has the second-largest economy in the world.

Daily data were converted into weekly data using the Wednesday-to-Wednesday values. Although a few studies, such as Arshanapalli and Doukas (1993), Beine et al. (2010), Junior et al. (2021), and Liu et al. (2017) use daily international data, some of them recognize the non-synchronous trading problem arising from different time zones (for instance, Tokyo and Shanghai are 14 and 12 h ahead of New York, respectively). Most studies that consider the US and Asia–Pacific countries use monthly data (e.g., Asafo-Adjei et al. 2021; Nyberg and Pönkä 2016; Rapach et al. 2013, 2015, 2019; Roll 1992; Tse 2018; Wen et al. 2015). The lead-lag effects tend to increase with data frequency; hence, if daily data is synchronous, it will present larger auto- and cross-correlations because there is better information to compensate for the rapid fluctuations of financial information. However, non-synchronous trading induces potentially serious biases in moments and co-moments (Lo and MacKinlay 1990a; Berben and Jansen 2005). Therefore, we work with weekly data to avoid non-synchronous trading due to different time zones while trying to capture most of the lead-lag effects. Data were collected on Wednesdays (or the previous business day if Wednesday was a holiday) to avoid eventual weekend effects, minimize holidays in the sample (Smith et al 1993), and use the best weekday to capture cross-correlations (Baumöhl and Lyócsa 2012). Berben and Jansen (2005) and Smith et al. (1993) are some examples of research that use weekly data in this framework.

Table 1 shows the data availability, including the starting dates when the series did not cover the entire period. China has the shortest time series as most of the series started after 1993, and the technology sector only started in 2015. Nevertheless, we chose to include China in our study because it is the largest and one of the fastest-growing emerging economies worldwide. Germany also presents two series that began quite late, but for the same reasons, we include this country in our study (the German market represents 2.5% of the world market value, according to the MSCI ACWI, on May 14, 2021).

**Table 1:** Data availability

	BM	CD	CS	EN	FI	HC	IN	RE	TEC	TEL	UT
Canada	C	C	C	C	C	C	C	01/07/1985	C	05/02/1976	C
France	C	C	C	C	C	C	C	C	C	17/10/1997	18/07/2000
Germany	C	C	C	03/05/2006	C	C	C	23/09/1993	03/11/1988	C	C
Japan	C	C	C	C	C	C	C	C	C	C	C
China	26/07/1993	26/07/1993	26/07/1993	02/12/1994	26/07/1993	27/02/2004	26/07/1993	26/07/1993	29/06/2015	15/11/2002	28/06/1995
UK	C	C	C	C	C	C	C	C	C	04/11/1981	05/12/1986
US	C	C	C	C	C	C	C	C	C	C	C

This table presents the availability of data for the 11 industries: Basic Materials (BM), Consumer Discretionary (CD), Consumer Staples (CS), Energy (EN), Financials (FI), Health Care (HC), Industrials (IN), Real Estate (RE), Technology (TEC), Telecommunications (TEL), and Utilities (UT). “C” indicates that the series is complete for the overall sample (01/01/1973 to 17/05/2021). The dates indicate the beginning of the series if they are only available after 01/01/1973

Table 2 contains the descriptive statistics of the weekly logarithmic returns of the 11 industries in the seven countries. While cross-industry variations are large, the table shows that, on average, the US presents the highest mean (0.13%) and the lowest risk level (2.76%). France offers the second-highest mean return (0.12%); however, it has a relatively high risk (3.35%). Canada, Japan, and the UK have the same mean returns (0.11%). The highest mean return is reported for the Canadian consumer staples sector (0.19%), and the lowest is for the German energy sector (− 0.05%). It should be noted that the data for this sector only started in 2006. The two sectors that present the highest trade-off between risk and return are consumer staples (CS), with an average return of 0.15% and a risk of 2.97%, and the health care (HC) sector, with an average of 0.15% and a risk of 2.90%.

**Table 2:** Descriptive statistics of weekly returns

	BM	CD	CS	EN	FI	HC	IN	RE	TEC	TEL	UT	Average
*Mean*												
Canada	0.07	0.10	0.19	0.08	0.14	0.14	0.14	0.07	0.11	0.12	0.09	0.11
France	0.17	0.15	0.18	0.11	0.12	0.15	0.16	0.09	0.15	0.00	− 0.01	0.12
Germany	0.10	0.14	0.17	− 0.05	0.08	0.12	0.10	0.10	0.20	0.02	0.07	0.09
Japan	0.09	0.13	0.13	0.07	0.07	0.15	0.13	0.10	0.13	0.18	0.07	0.11
China	0.07	0.16	0.10	0.05	0.10	0.18	0.06	0.18	− 0.02	0.02	0.05	0.09
UK	0.10	0.10	0.14	0.10	0.08	0.14	0.12	0.06	0.15	0.10	0.08	0.11
US	0.13	0.15	0.16	0.10	0.14	0.18	0.16	0.13	0.17	0.08	0.07	0.13
Average	0.10	0.13	0.15	0.07	0.10	0.15	0.12	0.10	0.13	0.07	0.06	0.11
*Std*												
Canada	3.66	2.81	2.29	3.65	2.69	4.13	3.35	2.71	4.31	2.56	2.21	3.12
France	3.21	3.52	3.07	3.86	3.61	2.99	3.45	2.75	4.41	3.19	2.78	3.35
Germany	2.95	3.86	2.96	3.25	3.24	2.37	3.07	2.77	3.82	3.71	2.56	3.14
Japan	3.23	2.83	2.92	4.10	3.50	2.59	3.06	3.99	3.73	4.20	3.33	3.41
China	4.43	4.62	4.75	3.91	3.79	3.12	3.96	4.90	1.72	2.68	3.61	3.77
UK	3.94	3.28	2.70	3.65	3.39	2.81	3.30	3.85	4.18	3.37	2.29	3.34
US	3.10	2.66	2.10	3.15	2.91	2.26	2.68	3.57	3.31	2.49	2.10	2.76
Average	3.50	3.37	2.97	3.65	3.30	2.90	3.27	3.51	3.64	3.17	2.70	3.27
*Skew*												
Canada	− 0.58	− 1.61	− 0.30	− 1.27	− 0.49	− 1.75	− 0.52	− 2.48	− 0.81	− 0.58	− 1.01	− 1.04
France	− 0.47	0.04	0.91	− 0.15	− 0.41	− 0.28	− 1.07	− 1.41	− 0.27	− 0.38	− 1.79	− 0.48
Germany	− 0.63	3.25	7.69	− 1.17	− 0.76	− 0.51	− 0.88	− 0.60	− 0.39	− 0.12	− 0.71	0.47
Japan	− 0.09	− 0.12	− 0.06	0.13	0.20	0.02	− 0.21	0.07	− 0.03	0.79	0.31	0.09
China	0.10	0.41	1.60	0.24	0.40	0.17	0.39	0.07	− 0.49	0.13	− 0.74	0.21
UK	− 0.49	− 0.78	− 0.18	− 0.33	− 0.45	− 0.11	− 0.68	− 0.50	− 0.24	0.05	− 0.06	− 0.34
US	− 0.66	− 0.59	− 0.60	− 1.11	− 0.70	− 0.48	− 0.75	− 0.43	− 0.51	− 0.42	− 0.60	− 0.62
*Kurt*												
Canada	7.71	29.72	5.93	17.14	8.82	27.60	6.04	33.10	10.98	9.86	12.00	15.35
France	5.79	11.86	19.65	13.10	9.07	4.78	13.82	25.91	6.89	13.42	26.16	13.68
Germany	7.42	81.84	217.5	23.00	13.29	6.65	10.40	21.27	11.84	6.72	8.66	37.14
Japan	6.40	5.45	5.63	5.51	7.18	5.01	5.18	6.90	5.41	7.43	7.77	6.17
China	10.01	14.27	24.84	11.52	13.72	22.53	10.81	14.95	38.70	15.05	13.17	17.23
UK	8.11	14.44	6.53	15.23	9.91	6.37	9.72	11.42	10.75	7.24	7.68	9.76
US	7.82	7.00	6.34	17.06	10.26	5.83	7.64	9.58	6.14	7.85	9.05	8.60

This table presents the mean, standard deviation (Std), skewness (Skew), and kurtosis (Kurt) of weekly logarithmic returns of the 11 industries (Basic Materials (BM), Consumer Discretionary (CD), Consumer Staples (CS), Energy (EN), Financials (FI), Health Care (HC), Industrials (IN), Real Estate (RE), Technology (TEC), Telecommunications (TEL), and Utilities (UT)) for Canada, France, Germany, Japan, China, the UK, and the US. Data cover different periods, all ending on 17/05/2021 (see Table 1). Mean and standard deviation values are in percentage

On average, the skewness was moderate across all countries. Weekly returns are left-skewed for Canada, France, the UK, and the US. All countries present excess kurtosis, especially Germany, mainly due to the consumer staple (CS) and consumer discretionary (CD) industries.

**Fig. 1:**
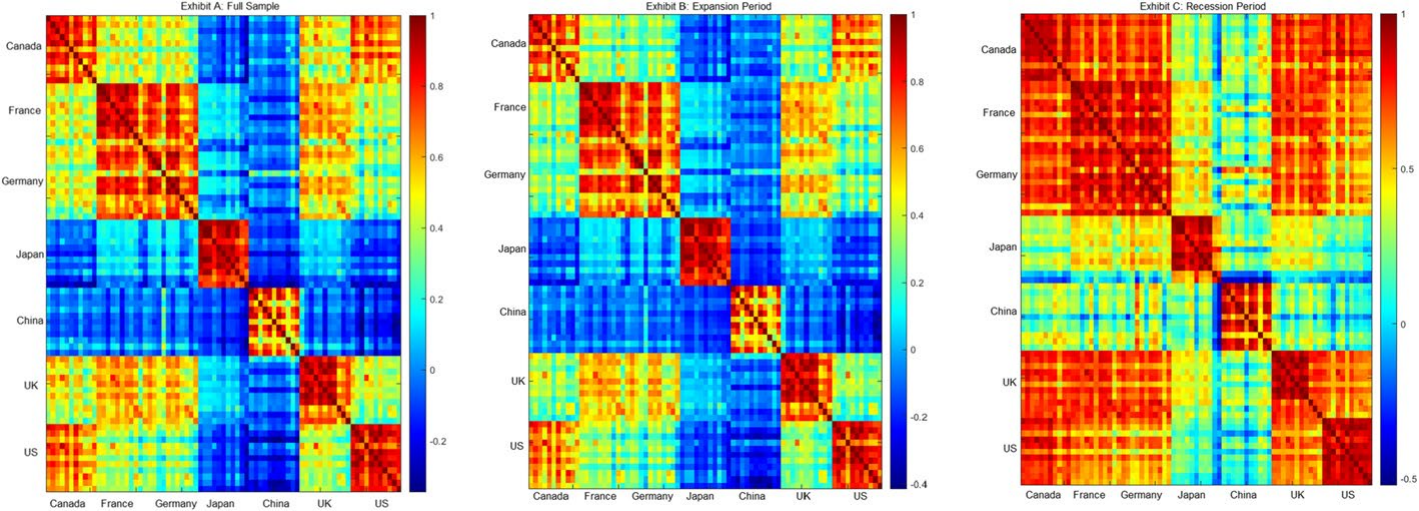
Correlation maps. *Notes*: This figure shows the correlation heat maps between the industries of 7 countries, for the full sample (Exhibit A), in expansion periods (Exhibit B), and recession periods (Exhibit C) in the US according to the NBER (https://www.nber.org/research/data/us-business-cycle-expansions-and-contractions). The countries are Canada, France, Germany, Japan, the UK, and the US. The industries, from the left (top) to the right (bottom), are Basic Materials (BM), Consumer Discretionary (CD), Consumer Staples (CS), Energy (EN), Financials (FI), Health Care (HC), Industrials (IN), Real Estate (RE), Technology (TEC), Telecommunications (TEL), and Utilities (UT). On the right side of each panel, the table reports the correlation scales associated with each color. The dark blue and dark red correspond to correlations of -0.5 and 1, respectively

Figure 1 shows the correlation maps for the series industry/country for the full sample period (Exhibit A), expansion periods (Exhibit B), and recession periods (Exhibit C). The partition of the data into expansion and recession periods is based on the National Bureau of Economic Research (NBER) business cycle classification in the US. For all samples, we can observe high and positive cross-country correlations between France and Germany and between Canada and the US. Interestingly, most industries in China and Japan are negatively correlated with industries in other countries in the overall sample and during the expansion periods. There are two main reasons for drawing from Fig. 1: first, as expected, correlations between industries of the same country present higher correlations, and second, correlations increase significantly during recession periods, especially between European and American countries.

## Methodology

In this section, we present the econometric tests implemented in the empirical application. Our analysis of the lead-lag relationships in international industries proceeds as follows. First, we estimate a bivariate vector autoregressive model of order one, VAR(1), for all the series (11 industries, 7 countries). The use of VARs with only one lag was suggested by the Akaike and Schwartz information criteria (AIC and BIC). Using the estimated VAR(1), we compute pairwise Granger causality tests and feedback measures. Next, we partition the data into expansion and recession periods according to the business cycle classification for the US and conduct a similar analysis in these periods. In addition to the analyses of causality and feedback in the mean, using the same methodology, we also study causality and feedback in volatility for the overall sample and for the expansion and recession periods. Finally, we study the Granger causality in the distribution of returns and volatility.

### Granger causality

The lead-lag relationship was first identified via Granger causality tests (Granger 1969). To establish the general result, suppose that we have two time series of returns $r_{1,t}$ and $r_{2,t}$. Suppose that their dynamics follow a bivariate VAR(1); then:

1 $$\left[ {\begin{array}{*{20}c} {{ }r_{1,t} } \\ {r_{2,t} } \\ \end{array} } \right] = \left[ {\begin{array}{*{20}c} {a_{1} } \\ {a_{2} } \\ \end{array} } \right] + \left[ {\begin{array}{*{20}c} {\phi_{1} } & {\phi_{1,2} } \\ {\phi_{2,1} } & {\phi_{2} } \\ \end{array} } \right]\left[ {\begin{array}{*{20}c} {{ }r_{1,t - 1} } \\ { r_{2,t - 1} } \\ \end{array} } \right] + \left[ {\begin{array}{*{20}c} {\varepsilon_{{r_{1} ,t}} } \\ {\varepsilon_{{r_{2} ,t}} } \\ \end{array} } \right],$$

2 $${{\varvec{\Omega}}} = Cov\left[ {\begin{array}{*{20}c} {\varepsilon_{{r_{1} t}} } \\ {\varepsilon_{{r_{2} ,t}} } \\ \end{array} } \right] = \left[ {\begin{array}{*{20}c} {\sigma_{{r_{1,t} }}^{2} } & {\sigma_{{r_{1,t} ,r_{2,t} }}^{2} } \\ {\sigma_{{r_{2,t} ,r_{1,t} }}^{2} } & {\sigma_{{r_{2,t} }}^{2} } \\ \end{array} } \right].$$

where $${{\varvec{\Phi}}} = \left[ {\begin{array}{*{20}c} {\phi_{1} } & {\phi_{1,2} } \\ {\phi_{2,1} } & {\phi_{2} } \\ \end{array} } \right]$$ is the coefficient matrix. It is common to assume that innovations are Gaussian and serially uncorrelated.

To assess the causality from $r_{2}$ to $r_{1}$ we test the hypotheses $\phi_{1,2} = 0$. Similarly, $r_{1}$ does not Granger cause $r_{2}$ if $\phi_{2,1} = 0$. The absence of Granger causality in either direction implies that coefficient matrix ${{\varvec{\Phi}}}$ is diagonal. Under this hypothesis, VAR(1) simplifies to:

3 $$\left[ {\begin{array}{*{20}c} {{ }r_{1,t} } \\ {r_{2,t} } \\ \end{array} } \right] = \left[ {\begin{array}{*{20}c} {a_{1} } \\ {a_{2} } \\ \end{array} } \right] + \left[ {\begin{array}{*{20}c} {\phi_{1} } & 0 \\ 0 & {\phi_{2} } \\ \end{array} } \right]\left[ {\begin{array}{*{20}c} {{ }r_{1,t - 1} } \\ { r_{2,t - 1} } \\ \end{array} } \right] + \left[ {\begin{array}{*{20}c} {\xi_{{r_{1} ,t}} } \\ {\xi_{{r_{2} ,t}} } \\ \end{array} } \right].$$

In the present case of VAR(1), testing Granger causality from $r_{2}$ to $r_{1}$ requires computing the sum of squared residuals of the regression of $r_{1,t} { }$ on $r_{1,t - 1}$, $RSS_{0}$, computing the sum of squared residuals of the regression of $r_{1,t}$ on $r_{1,t - 1}$ and $r_{2,t - 1}$, $RSS_{1}$, which are then used to compute:

4 $$S = \frac{{\left( {RSS_{1} - RSS_{0} } \right)}}{{RSS_{0} /\left( {T - 3} \right)}}.$$

Test $S$ follows an $F$ distribution with 1 and $T - 3$ degrees of freedom, that is, $F\left( {1,T - 3} \right)$. The Granger causality from $r_{1}$ to $r_{2}$ was tested analogously.

### Geweke measures of feedback

To assess information transmission between returns, we used Geweke’s (1982) feedback measures, which were applied to each industry/country return pair. They can be used to test the degree of feedback in both directions, contemporaneously and overall linear dependence.

Measure of lagged feedback from $r_{1}$ to $r_{2} :$

5 $$F_{{r_{1} \to r_{2} }} = \ln \left( {\frac{{\sigma_{{{\varvec{\xi}}_{{r_{2} }} }}^{2} { }}}{{\sigma_{{\varepsilon_{{r_{2} }} }}^{2} { }}}} \right).$$

Measure of lagged feedback from $r_{2}$ to $r_{1}$:

6 $$F_{{r_{2} \to r_{1} }} = \ln \left( {\frac{{\sigma_{{{\varvec{\xi}}_{{r_{1} }} }}^{2} { }}}{{\sigma_{{\varepsilon_{{r_{1} }} }}^{2} { }}}} \right).$$

Measure of contemporaneous feedback between $r_{1}$ and $r_{2}$:

7 $$F_{{r_{1} \leftrightarrow r_{2} }} = \ln \left( {\frac{{\sigma_{{\varepsilon_{{r_{1} }} }}^{2} \sigma_{{\varepsilon_{{r_{2} }} }}^{2} }}{{\left| {{\varvec{\Omega}}} \right|}}} \right).$$

Measure of total feedback (total linear dependence) between $r_{1}$ and $r_{2}$:

8 $$F_{{r_{1} .r_{2} }} = \ln \left( {\frac{{\sigma_{{{\varvec{\xi}}_{{r_{1} }} }}^{2} \sigma_{{{\varvec{\xi}}_{{r_{2} }} }}^{2} }}{{\left| {{\varvec{\Omega}}} \right|}}} \right).$$

$\left| {{\varvec{\Omega}}} \right|$ denotes the determinant of the innovation covariance matrix in the unrestricted model (Eq. 2). Under the null hypotheses, these measures, multiplied by the number of observations $T$, are asymptotically independent and follow chi-squared distributions with degrees of freedom $1$, $1$, $1,$ and $3$, respectively.

Because the feedback measures are only log-likelihood ratio statistics under the null hypotheses, their asymptotic distributions are well defined. In addition, under the alternative hypotheses, these measures, multiplied by the number of observations, asymptotically follow noncentral chi-squared distributions.

9 $$T\hat{F}_{{r_{1} \to r_{2} }} \sim {\mathcal{X}^{\prime}}^{2} \left( {1,TF_{{r_{1} \to r_{2} }} } \right),$$

10 $$T\hat{F}_{{r_{2} \to r_{1} }} \sim {\mathcal{X}^{\prime}}^{2} \left( {1,TF_{{r_{2} \to r_{1} }} } \right),$$

11 $$T\hat{F}_{{r_{1} \leftrightarrow r_{2} }} \sim {\mathcal{X}^{\prime}}^{2} \left( {1,TF_{{r_{1} \leftrightarrow r_{2} }} } \right),\quad {\text{and}}$$

12 $$T\hat{F}_{{r_{1} .r_{2} }} \sim {\mathcal{X}^{\prime}}^{2} \left( {3,TF_{{r_{1} .r_{2} }} } \right).$$

The measures presented above are additive, that is, $F_{{r_{1} .r_{2} }} = F_{{r_{1} \to r_{2} }} + F_{{r_{1} \leftrightarrow r_{2} }} + F_{{r_{2} \to r_{1} }} .$

### Granger causality in distribution

The test for Granger causality in mean and volatility offers an incomplete picture of the relationship between the conditional distributions of economic series, especially when these are fat-tailed, as expected for financial time series (Jeong et al. 2012). Several authors have proposed alternative tests that focus on the dependence of the quantiles of conditional distributions (e.g., Jeong et al. 2012; Hong et al. 2009). However, most of these tests aim to assess the dependence between only one quantile of a series at a time. Candelon and Tokpavi (2016) developed an alternative test that allows for the simultaneous consideration of several quantiles of distributions, thus increasing its power.

This subsection presents the test of Granger causality in distribution proposed by Candelon and Tokpavi (2016), from which the following description is heavily drawn. This test is based on value-at-risk (VaR), which is often used to assess the extent of loss of an asset or portfolio over a specific time frame. Considering $r_{i} = r_{1}$, $r_{2}$, the VaR at the $\alpha \%$ confidence level is given by:

13 $${\text{Pr}}[r_{i,t} < VaR_{t}^{{r_{i} }} \left( {{{\varvec{\uptheta}}}_{{r_{i} }}^{0} } \right){|}\space{\mathcal{F}}_{t - 1}^{{r_{i} }} {]} = \alpha .$$

$VaR_{t}^{{r_{i} }}$ is the VaR of asset $i$ at time $t,$ ${{\varvec{\uptheta}}}_{{r_{i} }}^{0}$ is the vector of true unknown finite-dimensional parameters related to the specification of the VaR models for $r_{i}$, and ${\mathcal{F}}_{t - 1}^{{r_{i} }}$. is the information set at time $t - 1$, defined as ${\mathcal{F}}_{t - 1}^{{r_{i} }} = \left[ {r_{i,l} , l \le t - 1} \right]$.

For each return series, a vector of VaRs at time $t$ based on the previous equation may be defined as follows: First, let $A$ be a set of risk levels, such that $A = \left\{ {\alpha_{1} ,\alpha_{2} , \ldots , \alpha_{m + 1} } \right\}$, for $0 < \alpha_{1} < \alpha_{2} < ... \alpha_{m + 1} < 1,$ which gives us

14 $$VaR_{t,1}^{{r_{i} }} \left( {{{\varvec{\uptheta}}}_{{r_{i} }}^{0} ,\alpha_{1} } \right) < VaR_{t,2}^{{r_{i} }} \left( {{{\varvec{\uptheta}}}_{{r_{i} }}^{0} ,\alpha_{2} } \right) < \ldots < VaR_{t,m + 1}^{{r_{i} }} \left( {{{\varvec{\uptheta}}}_{{r_{i} }}^{0} ,\alpha_{m + 1} } \right).$$

Next, the variables $r_{i,t}$ are divided into $m$ disjoint regions according to indicator variables that identify the events covering two consecutive VaR levels.

15 $$Z_{t,s}^{{r_{i} }} \left( {{{\varvec{\uptheta}}}_{{r_{i} }}^{0} } \right) = \left[ {\begin{array}{*{20}c} 1 & { if VaR_{t,s}^{{r_{i} }} \left( {{{\varvec{\uptheta}}}_{{r_{i} }}^{0} ,\alpha_{s} } \right) \le r_{i,t} < VaR_{t,s + 1}^{{r_{i} }} \left( {{{\varvec{\uptheta}}}_{{r_{i} }}^{0} ,\alpha_{s + 1} } \right)} \\ 0 & { otherwise } \\ \end{array} } \right. for \ s = 1, 2, \ldots, m.$$

Hence, to test the Granger causality in the distribution, we first define ${\mathbf{H}}_{t}^{{r_{i} }}$ as the vector containing the $m$ indicator variables, as defined in Eq. (15).

16 $${\mathbf{H}}_{t}^{{r_{i} }} \left( {{{\varvec{\uptheta}}}_{{r_{i} }}^{0} } \right) = \left( {Z_{t,1}^{{r_{i} }} \left( {{{\varvec{\uptheta}}}_{{r_{i} }}^{0} } \right), Z_{t,2}^{{r_{i} }} \left( {{{\varvec{\uptheta}}}_{{r_{i} }}^{0} } \right), \ldots , Z_{t,m}^{{r_{i} }} \left( {{{\varvec{\uptheta}}}_{{r_{i} }}^{0} } \right)} \right)^{T} .$$

Formally, $r_{2,t}$ does not Granger cause $r_{1,t}$ in the distribution if the following hypothesis holds:

17 $${\mathbb{H}}_{0} :{\mathbb{E}}\left[ {{\mathbf{H}}_{t}^{{r_{1} }} \left( {{{\varvec{\uptheta}}}_{{r_{1} }}^{0} } \right)|{\mathcal{F}}_{t - 1}^{{r_{1} \& r_{2} }} } \right] = {\mathbb{E}}\left[ {{\mathbf{H}}_{t}^{{r_{1} }} \left( {{{\varvec{\uptheta}}}_{{r_{1} }}^{0} } \right)|\space{\mathcal{F}}_{t - 1}^{{r_{1} }} } \right],$$

where ${\mathcal{F}}_{t - 1}^{{r_{1} \& r_{2} }} = \left\{ {\left( {r_{1,l} ,r_{2,l} } \right),l \le t - 1} \right\}$. Under the null hypothesis, the information set related to variable $r_{2t}$ does not provide any additional information to predict ${\mathbf{H}}_{t}^{{r_{1} }} \left( {{{\varvec{\uptheta}}}_{{r_{1} }}^{0} } \right)$ beyond the information present in the distribution support of $r_{1,t} .$

To test the hypothesis presented in Eq. (17), we estimate the conditional autoregressive value-at-risk (CAViaR) proposed by Engle and Manganelli (2004) for each series and risk level, which is defined as follows:

18 $$VaR_{t,s}^{{r_{i} }} \left( {{{\varvec{\uptheta}}}_{{r_{i} }}^{0} ,\alpha_{s} } \right) = \theta_{s0}^{{r_{i} }} + \theta_{s1}^{{r_{i} }} VaR_{t - 1}^{{r_{i} }} \left( {{{\varvec{\uptheta}}}_{{r_{i} }}^{0} } \right) + \theta_{s2}^{{r_{i} }} r_{i,t - 1}^{ + } + \theta_{s3}^{{r_{i} }} r_{i,t - 1}^{ - } + \varepsilon_{t,s} ,$$

where $r_{i,t - 1}^{ + } = {\text{max}}\left( {r_{i,t - 1} ,0} \right)$, $r_{i,t - 1}^{ - } = - {\text{min}}\left( {r_{i,t - 1} ,0} \right)$, and $s$ is the risk level. The error terms $\varepsilon_{t,s}$, conditional on all past information, form a stationary process with a continuous conditional density. The parameters of these CAViaR models were estimated using a quantile regression. Using the estimated VaRs, the empirical counterparts of ${\mathbf{H}}_{t}^{{r_{i} }}$ can be computed to obtain ${\hat{\mathbf{H}}}_{t}^{{r_{i} }} \equiv H_{t}^{{r_{i} }} \left( {\hat{\theta }_{1}^{{r_{i} }} , \ldots ,\hat{\theta }_{m}^{{r_{i} }} } \right)$.

The test statistic is obtained through the following four steps:

First, the sample cross-covariance matrix between ${\hat{\mathbf{H}}}_{t}^{{r_{1} }}$ and ${\hat{\mathbf{H}}}_{t}^{{r_{2} }}$ is computed as follows:

19 $${\hat{\mathbf{\Lambda }}}\left( j \right) \equiv \left[ {\begin{array}{*{20}c} {T^{ - 1} \mathop \sum \limits_{t = 1 + j}^{T} \left( {{\hat{\mathbf{H}}}_{t}^{{r_{1} }} - {\hat{\mathbf{\Pi }}}_{{r_{1} }} } \right)\left( {{\hat{\mathbf{H}}}_{t - 1}^{{r_{2} }} - {\hat{\mathbf{\Pi }}}_{{r_{2} }} } \right)^{T} } \ for & { 0 \le j \le T - 1} \\ {T^{ - 1} \mathop \sum \limits_{t = 1 - j}^{T} \left( {{\hat{\mathbf{H}}}_{t + j}^{{r_{1} }} - {\hat{\mathbf{\Pi }}}_{{r_{1} }} } \right)\left( {{\hat{\mathbf{H}}}_{t}^{{r_{2} }} - {\hat{\mathbf{\Pi }}}_{{r_{2} }} } \right)^{T} } \ for& { 1 - T \le j \le 0 } \\ \end{array} ,} \right.$$

where ${\hat{\mathbf{\Pi }}}_{{r_{1} }}$ and ${\hat{\mathbf{\Pi }}}_{{r_{2} }}$ represent the sample means for ${\hat{\mathbf{H}}}_{t}^{{r_{1} }}$ and ${\hat{\mathbf{H}}}_{t}^{{r_{2} }}$, respectively. Second, the corresponding sample cross-covariance matrix is computed as

20 $${\hat{\mathbf{R}}}\left( j \right) = diag({\hat{\mathbf{\Sigma }}}_{{r_{1} }} )^{{ - \frac{1}{2}}} {\hat{\mathbf{\Lambda }}}\left( j \right)diag({\hat{\Sigma }}_{{r_{2} }} )^{ - 1/2} ,$$

where $diag\left( . \right)$ is the diagonal form of matrix, and ${\hat{\mathbf{\Sigma }}}_{{r_{1} }}$ and ${\hat{\mathbf{\Sigma }}}_{{r_{2} }}$ are the sample covariance matrices of ${\hat{\mathbf{H}}}_{t}^{{r_{1} }}$ and ${\hat{\mathbf{H}}}_{t}^{{r_{2} }}$, respectively. Third, the quadratic form that accounts for the dependence between the current values of ${\hat{\mathbf{H}}}_{t}^{{r_{1} }}$ and lagged values of ${\hat{\mathbf{H}}}_{t}^{{r_{2} }}$ is calculated by

21 $$\hat{{\mathcal{T}}} = \mathop \sum \limits_{j = 1}^{T - 1} \kappa^{2} \left( \frac{j}{M} \right)\hat{Q}\left( j \right),$$

where $\kappa$ is a kernel function, $M$ is a truncation parameter, and $\hat{Q}\left( j \right)$ is defined as

22 $$\hat{Q}\left( j \right) = Tvec({\hat{\mathbf{R}}}\left( j \right))^{T} \left( {{\hat{\mathbf{\Gamma }}}_{{r_{1} }}^{ - 1} \otimes {\hat{\mathbf{\Gamma }}}_{{r_{2} }}^{ - 1} } \right)vec\left( {{\hat{\mathbf{R}}}\left( j \right)} \right),$$

where ${\hat{\mathbf{\Gamma }}}_{{r_{1} }}$ and ${\hat{\mathbf{\Gamma }}}_{{r_{2} }}$ represent the sample correlation matrices of ${\hat{\mathbf{H}}}_{t}^{{r_{1} }}$ and ${\hat{\mathbf{H}}}_{t}^{{r_{2} }}$, respectively. Lastly, the test statistic—a centered and scaled version of the quadratic form ${\hat{\mathcal{T}}}$—is given by

23 $$V_{Y \to X} = \frac{{\hat{{\mathcal{T}}} - m^{2} C_{T} \left( M \right)}}{{(m^{2} D_{T} \left( M \right))^{\frac{1}{2}} }}\to ^{d} {\mathcal{N}}\left( {0,1} \right),$$

where $C_{T} \left( M \right)$ and $D_{T} \left( M \right)$ are defined as

24 $$C_{T} \left( M \right){ } = \mathop \sum \limits_{j = 1}^{T - 1} (1 - \frac{j}{T})\kappa^{2} \left( \frac{j}{M} \right),$$

25 $$D_{T} \left( M \right) = 2\mathop \sum \limits_{j = 1}^{T - 1} (1 - \frac{j}{T})\left( {1 - \frac{j + 1}{T}} \right)\kappa^{4} \left( \frac{j}{M} \right).$$

In the empirical application we use $M = ln\left( T \right)$ and the Barlett kernel.^2^

### Out-of-sample performance evaluation

We complement the in-sample performance evaluation of our models by using out-of-sample tests. For each series, we use the last third of the sample as the testing period.

We use a recursive procedure to forecast the returns for every week of the out-of-sample period using the VAR model (Eqs. 1, 2). Then we assess its statistical performance using the R-squared out-of-sample

26 $$R_{OOS}^{2} = 1 - \frac{{\mathop \sum \nolimits_{t = \tau }^{T} \left( {r_{i,t} - \hat{r}_{i,t} } \right)^{2} }}{{\mathop \sum \nolimits_{t = \tau }^{T} \left( {r_{i,t} - \overline{r}_{i,t} } \right)^{2} }},$$

where $\tau$ denotes the first week of the out-of-sample period; ${\hat{r}}_{i,t}$ is the prediction of return $i$ during week t, and ${\overline{r}}_{i,t}$ is the return forecast based on the historical average. We assessed the statistical significance of the ${R}_{OOS}^{2}$ using the mean squared prediction error (MSPE) adjusted statistic proposed by Clark and West (2007).

The ultimate performance test of a model relies on its value for real-world investors. Thus, we design two long-short trading strategies based on return forecasts. First, for each country, the investor takes a long position in the industry with the highest predicted return and a short position in the industry with the lowest predicted return. Second, for each industry, the investor takes a long position in the non-US country that has the highest forecasted return and a short position in the non-US country with the lowest predicted return. After this, we compute the alphas of these strategies using as the market portfolio the MSCI World Total Return Index^3^ and as the risk-free rate the 4-week T-bill rate as the risk-free rate. Other trading strategies based on lead-lag information have also been presented in the literature, with positive results (see, for instance, Copeland and Copeland 1998; Haque 2011; Haque 2011; Stübinger 2019, and most notably, Li et al. 2022).

Finally, we analyze the economic value of our return and volatility forecasts for a constant relative risk aversion (CRRA) investor using certainty equivalent return (CER); that is, for each country and industry, each week the investor chooses what fraction of their wealth to invest in the industry portfolio and the risk-free rate^4^ based on the return and volatility forecasts. We then calculate the realized return series for the portfolios and compute the CER of an investor with a relative risk aversion coefficient of 3.

## Empirical results

This section presents the results on causality and feedback between industry returns and volatility from and to the US and other countries: Canada (CN), France (FR), Germany (GE), Japan (JP), China (CH), and the UK; the causality in the mean during expansion and recession periods in the US; the causality in quantiles for returns and volatility; and finally, the out-of-sample performance of the models and long-short strategies.

### Causality and feedback in the mean

Table 3 shows the coefficients of the VAR(1) models and the significance of the Granger causality bivariate tests on returns considering the unrestricted and restricted VAR(1) models (Eqs. 1, 2, 3).

**Table 3:** Coefficients of the VAR(1) and significance of Granger causality in the mean

	BM	CD	CS	EN	FI	HC	IN	RE	TEC	TEL	UT
$US\to CN$	0.116*	0.161*	0.067*	0.095*	0.065*	0.103*	0.063	0.052*	− 0.004	0.072*	0.105*
$CN\to US$	− 0.044	− 0.024	0.037	0.032	0.004	− 0.017	0.005	− 0.019	0.061*	− 0.057*	**0.047**
$US\to FR$	0.099*	**0.061**	0.036	0.134*	**0.058**	0.024	0.033	0.043*	0.032	0.000	0.111*
$FR\to US$	− 0.044	− 0.024	0.037	0.032	0.004	− 0.017	0.005	− 0.019	0.061*	− 0.057	0.047
$US\to GE$	0.110*	0.117*	**0.069**	0.070*	**0.058**	0.076*	**0.067**	0.017	0.018	0.026	0.067*
$GE\to US$	− 0.002	− 0.014	0.021	0.009	− 0.033	0.028	− 0.015	− 0.002	0.064*	0.008	− 0.001
$US\to JP$	0.119*	0.128*	0.097*	0.145*	0.104*	0.137*	0.162*	0.100*	0.167*	0.115*	0.108*
$JP\to US$	− 0.019	− 0.052	− 0.013	0.022	**− 0.036**	− 0.017	− 0.019	− 0.001	− 0.013	− 0.023	0.013
$US\to CH$	0.188*	0.034	0.033	0.122*	0.051	**0.056**	0.057	0.024	0.004	0.058*	0.017
$CH\to US$	− 0.017	− 0.009	− 0.003	0.024	− 0.053*	0.022	− 0.026	− 0.024	0.040	− 0.013	− 0.003
$US\to UK$	0.144*	0.115*	0.090*	0.154*	0.089*	0.076*	0.092*	0.107*	0.117*	**0.059**	**0.045**
$UK\to US$	0.005	0.003	0.060*	0.076*	− 0.032	**0.046**	0.013	0.017	0.013	− 0.030	0.011

This table presents the coefficients of a bivariate VAR(1) and the significance of the Granger causality tests resulting from a bivariate VAR(1) using weekly returns. Data cover different periods, all ending on 17/05/2021 (see Table 1). The tests are conducted pairwise between the US and other 6 countries (Canada (CN), France (FR), Germany (GE), Japan (JP), China (CH), and the UK) for 11 industries (Basic Materials (BM), Consumer Discretionary (CD), Consumer Staples (CS), Energy (EN), Financials (FI), Health Care (HC), Industrials (IN), Real Estate (RE), Technology (TEC), Telecommunications (TEL), and Utilities (UT)). $US\to k$ indicates Granger causality from the returns of US to the returns of country $k$, and $k\to US$ indicates Granger causality from country $k$ to the US, for each industry. Numbers in bold indicate the rejection of the null hypothesis of no Granger causality at the 5% level. One asterisk, “*”, denotes significance at the 1% level

The results in Table 3 show that the US plays a leading role within each industry. From the 66 causality tests $US \to k$, 49 are significant at the 5% level, from which 41 present a *p* value less than 1%, while from the other 66 causality tests $k \to US$, only 11 are significant at the 5% level. All VAR(1) coefficients for $US \to k$ (except for $US \to CN$, which is not significant) are positive. Only four coefficients for $k \to US$ are higher than $US \to k$, namely for Canada, France, and Germany in the telecommunications (TEC) industry and China in financials (FI). Therefore, there is evidence that causality may also run from these countries to the US. The negative sign of the causality coefficient from China to the US may indicate that Chinese financials have been taking a larger share worldwide at the expense of the US sector. Overall, these results suggest that lagged returns in US industries contain relevant information to predict the returns of other countries’ industries. This is especially evident in Japan, the UK, and Canada.

Table 4 reports the estimated pairwise Geweke feedback measures between countries within each industry. The results indicate that there is linear dependence between the US and the other countries for all industries. The contemporaneous feedback is the major contributor to the total feedback, where the percentages range from 72%, for utilities (UT) in Japan, to 99.5%, for industrials (IN) in France, with an overall average value of 94.1%. These results suggest that most markets are highly integrated and that, on average, 94.1% of the return variability is transmitted within 1 week. The level of integration is weaker for Asian markets, which present the lowest values for the contemporaneous feedback. Arguably, the level of market integration worldwide (contemporaneous feedback) has been increasing in the last 40 years due to the ongoing globalization process. Most countries have become increasingly integrated, both in terms of real and financial transactions, especially EU countries owing to the introduction of the Euro (Beine et al. 2010). Significantly higher international correlations mean that international stock market spillovers have also become more significant as the link between stock market and real economy has intensified; for example, because of greater household shareholdings (Berben and Jansen 2005).

**Table 4:** Geweke feedback measures in the mean

	BM	CD	CS	EN	FI	HC	IN	RE	TEC	TEL	UT
$${F}_{US\to k}$$											
CN	0.007^*^	0.016^*^	0.004^*^	**0.003**	**0.003**	0.005^*^	0.004^*^	0.005^*^	0.000	0.004^*^	0.008^*^
	*1.0%*	*3.8%*	*1.9%*	*0.5%*	*0.5%*	*2.7%*	*0.6%*	*2.4%*	*0.0%*	*3.2%*	*3.8%*
FR	0.007^*^	**0.003**	**0.002**	0.009^*^	**0.002**	0.002	0.001	**0.003**	0.001	0.000	0.006^*^
	*2.0%*	*1.2%*	*1.5%*	*2.4%*	*0.6%*	*0.9%*	*0.3%*	*3.3%*	*0.2%*	*0.0%*	*4.2%*
GE	0.009^*^	0.009^*^	0.004^*^	0.005^*^	**0.002**	0.009^*^	**0.003**	0.001	0.000	0.001	**0.003**
	*2.5%*	*5.1%*	*6.6%*	*3.9%*	*0.7%*	*5.2%*	*1.0%*	*0.9%*	*0.1%*	*0.6%*	*3.3%*
JP	0.012^*^	0.015^*^	0.005^*^	0.012^*^	0.007^*^	0.014^*^	0.020^*^	0.008^*^	0.020^*^	0.005^*^	0.006^*^
	*9.7%*	*10.1%*	*7.7%*	*10.4%*	*7.0%*	*19.2%*	*12.1%*	*15.9%*	*12.4%*	*13.9%*	*26.0%*
CH	0.016^*^	0.000	0.000	0.009^*^	0.002	0.002	0.001	0.000	0.000	**0.003**	0.000
	*12.9%*	*1.2%*	*4.2%*	*10.1%*	*3.0%*	*7.4%*	*3.2%*	*4.1%*	*0.8%*	*8.5%*	*1.3%*
UK	0.009^*^	0.008^*^	0.005^*^	0.011^*^	0.005^*^	0.004^*^	0.007^*^	0.009^*^	0.008^*^	**0.002**	**0.003**
	*1.9%*	*3.3%*	*2.5%*	*1.9%*	*1.2%*	*1.5%*	*2.2%*	*8.4%*	*3.9%*	*1.4%*	*2.6%*
$${F}_{k\to US}$$											
CN	0.001	0.001	0.001	0.001	0.000	0.001	0.001	0.000	0.005^*^	**0.003**	**0.002**
	*0.2%*	*0.2%*	*0.6%*	*0.2%*	*0.0%*	*0.7%*	*0.2%*	*0.1%*	*1.2%*	*2.0%*	*1.1%*
FR	0.000	0.001	0.002	0.000	0.001	0.001	0.000	0.000	0.004^*^	0.001	0.001
	*0.1%*	*0.5%*	*1.1%*	*0.1%*	*0.4%*	*0.4%*	*0.1%*	*0.1%*	*1.6%*	*0.6%*	*0.7%*
GE	0.000	0.001	0.002	0.000	0.001	0.001	0.000	0.001	0.004^*^	0.000	0.000
	*0.0%*	*0.3%*	*2.8%*	*0.4%*	*0.4%*	*0.4%*	*0.1%*	*0.8%*	*2.0%*	*0.2%*	*0.2%*
JP	0.000	0.003	**0.000**	0.001	0.002	0.000	0.001	0.001	0.000	0.002	0.000
	*0.2%*	*1.9%*	*0.6%*	*1.0%*	*1.8%*	*0.6%*	*0.4%*	*1.9%*	*0.2%*	*4.5%*	*2.0%*
CH	0.001	0.000	0.001	0.001	0.005^*^	0.001	0.001	0.001	0.001	0.001	0.000
	*0.7%*	*1.5%*	*6.2%*	*1.5%*	*8.6%*	*5.9%*	*3.2%*	*11.1%*	*5.2%*	*3.6%*	*0.6%*
UK	0.000	0.001	0.006^*^	0.005^*^	0.002	**0.003**	0.000	0.000	0.000	0.002	0.000
	*0.0%*	*0.4%*	*3.2%*	*0.9%*	*0.4%*	*1.3%*	*0.2%*	*0.4%*	*0.3%*	*1.1%*	*0.4%*
$${F}_{US\leftrightarrow k}$$											
CN	0.729^*^	0.407^*^	0.195^*^	0.729^*^	0.591^*^	0.170^*^	0.584^*^	0.219^*^	0.397^*^	0.134^*^	0.207^*^
	*98.8%*	*95.9%*	*97.5%*	*99.3%*	*99.5%*	*96.6%*	*99.2%*	*97.5%*	*98.8%*	*94.8%*	*95.1%*
FR	0.325^*^	0.259^*^	0.141^*^	0.367^*^	0.354^*^	0.194^*^	0.304^*^	0.095^*^	0.251^*^	0.110^*^	0.142^*^
	*97.9%*	*98.3%*	*97.4%*	*97.5%*	*99.0%*	*98.7%*	*99.5%*	*96.5%*	*98.2%*	*99.4%*	*95.1%*
GE	0.366^*^	0.166^*^	0.055^*^	0.122^*^	0.327^*^	0.167^*^	0.339^*^	0.068^*^	0.219^*^	0.115^*^	0.083^*^
	*97.5%*	*94.6%*	*90.7%*	*95.7%*	*98.9%*	*94.4%*	*99.0%*	*98.3%*	*97.9%*	*99.2%*	*96.5%*
JP	0.110^*^	0.130^*^	0.064^*^	0.099^*^	0.093^*^	0.059^*^	0.142^*^	0.041^*^	0.140^*^	0.029^*^	0.017^*^
	*90.1%*	*88.1%*	*91.7%*	*88.6%*	*91.2%*	*80.2%*	*87.5%*	*82.2%*	*87.4%*	*81.6%*	*72.0%*
CH	0.105^*^	0.033^*^	0.008^*^	0.078^*^	0.052^*^	0.020^*^	0.041^*^	0.009^*^	0.020^*^	0.030^*^	0.025^*^
	*86.3%*	*97.4%*	*89.6%*	*88.4%*	*88.4%*	*86.8%*	*93.6%*	*84.7%*	*94.0%*	*87.9%*	*98.1%*
UK	0.451^*^	0.238^*^	0.189^*^	0.569^*^	0.408^*^	0.242^*^	0.292^*^	0.099^*^	0.184^*^	0.162^*^	0.097^*^
	*98.0%*	*96.4%*	*94.3%*	*97.2%*	*98.4%*	*97.2%*	*97.7%*	*91.3%*	*95.8%*	*97.6%*	*97.0%*
$${F}_{US,k}$$											
CN	0.738^*^	0.424^*^	0.200^*^	0.734^*^	0.594^*^	0.176^*^	0.589^*^	0.225^*^	0.402^*^	0.141^*^	0.218^*^
FR	0.332^*^	0.264^*^	0.145^*^	0.376^*^	0.358^*^	0.196^*^	0.306^*^	0.099^*^	0.256^*^	0.110^*^	0.149^*^
GE	0.375^*^	0.175^*^	0.061^*^	0.128^*^	0.330^*^	0.177^*^	0.343^*^	0.069^*^	0.223^*^	0.116^*^	0.086^*^
JP	0.122^*^	0.148^*^	0.070^*^	0.111^*^	0.102^*^	0.074^*^	0.162^*^	0.050^*^	0.160^*^	0.036^*^	0.024^*^
CH	0.122^*^	0.033^*^	0.009^*^	0.088^*^	0.059^*^	0.023^*^	0.044^*^	0.011^*^	0.022^*^	0.034^*^	0.026^*^
UK	0.460^*^	0.247^*^	0.201^*^	0.586^*^	0.415^*^	0.249^*^	0.299^*^	0.109^*^	0.192^*^	0.166^*^	0.100^*^

This table presents the Geweke feedback measures resulting from a bivariate VAR(1). Data cover different periods, all ending on 17/05/2021 (see Table 1). The tests are conducted pairwise between the returns of the US and other six countries (Canada (CN), France (FR), Germany (GE), Japan (JP), China (CH), and the UK) for 11 industries (Basic Materials (BM), Consumer Discretionary (CD), Consumer Staples (CS), Energy (EN), Financials (FI), Health Care (HC), Industrials (IN), Real Estate (RE), Technology (TEC), Telecommunications (TEL) and Utilities (UT)). ${F}_{US\to k}$ is the measure of lagged feedback from the US to country $k$, ${F}_{k\to US}$ is the measure of lagged feedback from country $k$ to the US, ${F}_{US\leftrightarrow k}$ is the measure of contemporaneous feedback$,$ and ${F}_{US,k}$ is the measure of total feedback. Numbers in bold indicate the rejection of the null of no feedback at the 5% level. One asterisk, “*”, denotes significance at the 1% level. Numbers in italic represent the weight of the lagged and contemporaneous feedbacks to the total feedback

For all industries, the percentage of lagged feedback from the US to non-US countries is substantially higher than that in the opposite direction. Therefore, lagged feedback is asymmetrical and runs dominantly from the US to other countries, and, in most cases, is unidirectional. We highlight the results for lagged feedback from the US to Japan, which are significant at the 1% level and show high weights for all industries. Lagged feedback to Japan in utilities (UT) (26%) presents the highest value of lagged feedback across all countries and industries. Conversely, the lagged feedback from non-US countries to the US is marginal and, in most cases, not significant at the 5% level. We report a maximum significant relative value of 8.6% (0.005 in absolute terms) from China for the financials (FI).

Tables 3 and 4 also show that, at the industry level, US basic materials (BM) and energy (EN) show causality to all countries at the 1% significance level. This is possibly justified by the types of commodities produced by these industries, such as oil, metals, and coal, which are highly export-oriented and whose shocks have historically led the global economy into a downturn (Venditti and Veronese 2020). The lagged US returns also contain relevant information to predict the returns of non-US countries for financial (FI) returns, except for China. This is expected because of the high degree of financial sector integration worldwide and the fact that firms in many industries rely heavily on financial services and intermediaries. Therefore, they are expected to have a significant impact on companies worldwide (Rapach et al. 2015).

Table 5 presents the cross-industry coefficients of VAR(1) and the significance of Granger causality considering only the unidirectional relationship $US \to k$.

**Table 5:** Cross-industry coefficients of the VAR(1) and significance of Granger causality in the mean from the US to other countries

	BM	CD	CS	EN	FI	HC	IN	RE	TEC	TEL	UT
Panel A: Canada											
BM	0.116*	0.138*	0.022	0.111*	0.074*	0.084*	**0.063**	0.105*	0.005	0.072*	0.057*
CD	0.064	0.161*	0.030	**0.080**	0.082	0.065	0.058	0.087*	0.040	0.107*	0.045*
CS	0.064	0.176*	**0.067**	**0.084**	0.091	0.083	0.019	0.117*	0.037	0.085*	0.065*
EN	0.020	0.083*	0.015	0.095*	0.039	**0.066**	0.036	0.065*	− 0.005	0.044*	0.047*
FI	− 0.003	0.111*	0.020	0.049	0.065	**0.069**	0.031	0.080*	− 0.021	0.096*	0.046*
HC	0.030	0.148*	**0.049**	0.066	0.069	0.103*	0.028	0.091*	0.029	0.096*	**0.047**
IN	0.045	0.154*	0.032	**0.069**	0.068	**0.076**	0.063	0.083*	− 0.004	0.083*	0.046*
RE	0.022	0.083*	0.041*	0.057*	0.044	**0.052**	0.034	0.052*	− 0.013	0.037*	0.045*
TEC	0.010	0.090*	− 0.002	0.034	0.028	**0.056**	0.040	0.050*	− 0.004	0.064*	0.014
TEL	0.003	0.092*	0.006	0.022	0.024	0.045	0.014	0.045	0.055	0.072*	**0.044**
UT	0.048	0.085*	**0.051**	**0.035**	0.022	0.028	0.013	0.069*	0.025	0.073*	0.105*
Panel B: France											
BM	0.099*	0.045	0.006	**0.062**	**0.072**	0.027	**0.060**	**0.066**	0.026	− **0.054**	0.082*
CD	0.059	**0.061**	− 0.003	0.002	0.046	0.008	0.041	0.027	0.042	− 0.045	**0.045**
CS	0.061	**0.073**	0.036	0.015	**0.092**	0.039	**0.095**	**0.068**	0.048	− 0.039	0.076*
EN	0.072*	0.003	0.021	0.134*	0.078*	0.018	0.040	0.064*	0.036	− 0.026	0.088*
FI	0.023	0.005	− 0.004	− 0.008	0.058	0.012	0.006	0.051	0.011	− 0.047	0.036
HC	0.037	0.043	0.021	− 0.008	0.053	0.024	0.049	0.043	0.013	− 0.047	0.039
IN	0.041	0.018	− 0.007	0.015	0.057	0.005	0.033	0.050	− 0.013	**− 0.064**	**0.052**
RE	0.036	0.031	0.033	− 0.002	0.026	**0.043**	0.015	**0.043**	0.020	− 0.034	**0.032**
TEC	0.027	0.038	0.002	− 0.001	0.032	0.005	0.035	0.026	0.032	− 0.018	0.031
TEL	0.028	0.022	− 0.039	− 0.057	0.013	− 0.040	0.021	0.038	0.020	0.000	0.039
UT	**0.068**	0.059	0.061	0.039	0.044	**0.066**	0.085	0.095*	0.008	− 0.004	0.111*
Panel C: Germany											
BM	0.110*	0.105*	0.032	0.073*	0.071*	0.056*	0.100*	0.036	0.017	− 0.007	0.057*
CD	0.091*	0.117*	0.031	0.006	0.078*	**0.047**	**0.071**	**0.042**	0.020	0.003	**0.042**
CS	0.099*	0.113*	**0.069**	0.022	0.107*	0.097*	**0.081**	0.003	− 0.027	− 0.003	**0.052**
EN	0.078*	0.092*	0.034	0.070*	**0.060**	0.058*	0.065*	0.023	0.003	0.002	0.051*
FI	0.031	**0.065**	0.002	0.008	**0.058**	0.032	0.029	0.017	− 0.020	− 0.008	0.031
HC	**0.066**	**0.077**	0.033	0.007	0.060	**0.076**	0.053	0.003	− 0.040	− 0.013	**0.054**
IN	0.088*	0.107*	0.024	0.022	**0.067**	**0.052**	**0.067**	0.023	0.000	− 0.013	**0.041**
RE	**0.039**	**0.056**	0.013	0.016	**0.043**	**0.034**	0.021	0.017	− 0.021	− 0.014	0.041*
TEC	0.047	**0.055**	0.009	0.014	**0.043**	**0.031**	0.038	0.029	0.018	0.015	**0.042**
TEL	0.008	0.039	− 0.027	0.031	0.015	− 0.021	0.009	0.016	0.018	0.026	0.010
UT	**0.063**	0.057	0.030	0.059	0.064	**0.049**	0.036	0.030	− 0.032	0.001	**0.067**
Panel D: Japan											
BM	0.119*	0.099*	0.104*	0.147*	0.083*	0.068*	0.142*	0.087*	0.117*	0.034	0.026
CD	0.107*	0.128*	0.137*	0.131*	0.091*	0.077*	0.152*	**0.131**	0.160*	**0.067**	0.026
CS	0.115*	0.104*	0.097*	0.168*	0.104*	0.117*	0.115*	**0.185**	0.102*	**0.091**	**0.066**
EN	0.081*	0.047*	0.047*	0.145*	0.066*	0.058*	0.093*	**0.065**	0.090*	**0.054**	0.023
FI	0.087*	0.089*	0.091*	0.117*	0.104*	0.079*	0.113*	**0.141**	0.113*	**0.070**	**0.052**
HC	0.127*	0.109*	0.108*	0.171*	0.122*	0.137*	0.116*	**0.176**	0.120*	**0.088**	0.087*
IN	0.122*	0.128*	0.133*	0.153*	0.121*	0.085*	0.162*	**0.131**	0.166*	**0.076**	0.047
RE	0.069*	0.063*	0.066*	0.072*	0.075*	0.061*	0.077*	**0.100**	0.088*	0.070*	0.033
TEC	0.083*	0.099*	0.103*	0.080*	**0.059**	0.046*	0.124*	**0.086**	0.167*	**0.058**	0.021
TEL	0.083*	0.105*	0.105*	0.106*	0.099*	0.067*	0.119*	**0.136**	0.135*	0.115*	0.041
UT	0.101*	0.088*	0.083*	0.166*	**0.090**	0.118*	0.103*	**0.143**	0.123*	0.115*	0.108*
Panel E: China											
BM	0.188*	**0.083**	0.022	0.147*	0.133*	0.089*	0.097*	0.110*	0.019	0.080*	0.046
CD	0.118*	0.034	0.018	**0.070**	**0.072**	**0.065**	0.040	0.035	0.006	**0.046**	0.028
CS	0.052	− 0.016	0.033	0.059	0.047	**0.064**	0.010	0.016	0.022	**0.065**	0.019
EN	0.136*	0.057	0.019	0.122*	0.097*	0.078*	0.081*	0.121*	0.016	0.100*	0.069*
FI	0.067	− 0.019	0.035	0.055	0.051	**0.048**	0.014	0.056	0.009	0.020	0.026
HC	0.031	− 0.062	− 0.001	0.034	0.030	0.056	− 0.020	− 0.003	0.009	**0.057**	0.021
IN	0.112*	0.011	0.039	**0.110**	**0.109**	0.082*	0.057	0.059	0.006	0.057*	0.032
RE	**0.053**	− 0.002	0.011	0.023	0.020	0.050*	0.025	0.024	0.002	0.025	0.016
TEC	**0.056**	0.019	0.034	0.048	**0.051**	0.056*	0.024	0.015	0.004	**0.037**	0.013
TEL	0.111*	0.017	− 0.012	**0.070**	0.047	0.093*	0.024	0.011	0.007	0.058*	0.044
UT	0.134*	− 0.002	0.055	0.106*	**0.079**	**0.066**	0.081	0.070	0.001	0.097*	0.017
Panel F: UK											
BM	0.144*	0.100*	0.080*	0.109*	0.126*	0.080*	0.105*	0.102*	**0.063**	0.034	**0.038**
CD	0.103*	0.115*	0.045	0.058	0.118*	0.045	0.090*	0.087*	0.122*	0.052	0.012
CS	0.125*	0.118*	0.090*	**0.097**	0.135*	0.089*	0.101*	0.116*	0.080	0.068	0.034
EN	**0.075**	0.074*	0.064*	0.154*	0.104*	0.061*	0.090*	0.088*	**0.061**	0.075*	0.047*
FI	0.016	**0.060**	0.020	0.031	0.089*	0.037	0.027	**0.077**	0.047	0.043	0.016
HC	**0.089**	0.089*	**0.059**	0.046	0.109*	0.076*	0.059	**0.095**	**0.083**	0.051	0.038
IN	0.104*	0.107*	0.070*	0.091*	0.145*	0.075*	0.092*	0.099*	**0.083**	0.047	0.026
RE	0.047	0.057*	0.059*	0.037	0.084*	0.061*	0.064*	0.107*	0.024	0.016	0.025
TEC	**0.075**	0.080*	0.027	0.046	0.076*	0.035	0.066*	**0.066**	0.117*	0.031	0.011
TEL	0.063	**0.068**	− 0.004	0.042	0.055	0.008	0.050	0.050	0.058	**0.059**	− 0.018
UT	0.120*	**0.089**	0.088*	0.070	0.108*	0.082*	0.112*	0.106*	0.014	0.059	0.045

This table presents the coefficients of a bivariate VAR(1) and the significance of the Granger causality tests resulting from a bivariate VAR(1) using weekly returns. Data cover different periods, all ending on 17/05/2021 (see Table 1). The tests are conducted pairwise between cross-industry from the US to other countries. The industries are Basic Materials (BM), Consumer Discretionary (CD), Consumer Staples (CS), Energy (EN), Financials (FI), Health Care (HC), Industrials (IN), Real Estate (RE), Technology (TEC), Telecommunications (TEL), and Utilities (UT). Numbers in bold indicate the rejection of the null hypothesis of no Granger causality at the 5% level. One asterisk, “*”, denotes significance at the 1% level

From Table 5, we can observe that the US returns of the basic materials (BM) Granger cause, at the 5% significance level, 50 out of 66 series industry/country series; that is, causality runs in more than 75%, while the US returns of energy (EN) Granger cause 48 series, corresponding to more than 72% of the time. At the 1% significance level, these figures were reduced to 41 for both the BM and EN industries. Although there are other significant cross-industry linkages, BM and EN are the main sectors that transmit information to other counties. Most notably, Japan appears to be prominent as it has the most significant cross-industry relationship with the US'. These findings are highly plausible because firms in other industries rely heavily on commodities and fuel (Venditti and Veronese 2020; Khalfaoui et al. 2021). Furthermore, the lagged returns for commodity- and material-producing sectors placed earlier in the production chain are frequently strongly connected to the returns of industries positioned later in the chain (Rapach et al. 2015). This outcome is consistent with commodity positive price shocks that increase product prices and returns for industries in earlier stages of production, while reducing profit margins and causing lower returns for industries positioned in later production phases. Due to overall economic interdependence, a positive cash flow shock in one industry has implications for cash flows in other industries. However, information-processing limitations inhibit investors in other industries from quickly adjusting equity prices to the full impact of cash flows, leading to cross-industry return predictability.

In summary, our findings suggest that the US is the dominant market in terms of information transmission in most industries, except for China. The leading role of the US is justified by its economy being the world’s largest in terms of GDP and an important trading partner for many countries. Additionally, the US financial market exhibits the world’s largest market capitalization. According to data from the World Bank (2021), in 2019, the market capitalization of listed domestic companies was $33.89 trillion, approximately 41% of the worldwide total. Furthermore, the US market worldwide. For instance, US industry indices are often used as benchmarks in fixed-income markets because they offer both a great breadth of coverage and length of historical data. This high coverage and attention from investors and analysts gradually impact the macroeconomic fundamentals of the US market across international markets (Rizova 2010; Rapach et al. 2013).

Other possible explanations for the key role of the US industry may relate to institutional holdings, market share, and trading volume.

Badrinath et al. (1995) found that the firms’ institutional ownership influences their lead-lag role. This relates to the “prudent man” rule that governs the investment behavior of institutional portfolio managers. According to this rule, portfolio managers are required to make “cautious” investments. Consequently, institutional investors are compelled to invest in a subset of tradable assets. Badrinath et al. (1995) found that when firms are owned by institutions, they play a leading role over non-institutional firms. According to the World Bank (2021), in 2019, most firms in the US market were institutionally owned, which may influence the leading role of the country.

New information generally has a greater influence on industry leaders with large market shares. Because of market frictions, this information may not be immediately incorporated into the prices of other firms. As a result, there is a lead-lag relationship between industry leaders and followers. According to Lo and MacKinlay (1990b), Brennan et al. (1993), and Hou (2007), slow transmission of information can be attributed to a variety of factors, including incomplete markets and constrained stock market participation, information asymmetries, noise traders, limited investor attention, transaction costs, short sale constraints, legal constraints faced by institutional investors, and other forms of market frictions and institutional constraints.

Finally, Chordia and Swaminathan (2000) argue that trading volume is a key driver of the lead-lag pattern detected in stock markets as low-volume stocks tend to adjust more slowly to information than high-volume stocks. According to the World Bank (2021), in 2019 the US reported a stock trading volume of approximately 23.192 trillion dollars (the highest national value), while China reported 18.248 trillion dollars, Japan 5.097 trillion dollars, the UK 2.357 trillion dollars, Canada 1.432 trillion dollars, Germany 1.350 trillion dollars, and France 1.168 trillion dollars.

### Causality and feedback in volatility

This subsection examines the causal and lead-lag relationships between the volatilities of industries from and to the US and other countries (Canada, France, Germany, Japan, China, and the UK). The weekly series of volatilities is constructed using the standard deviation of daily returns within a week, measured on Wednesdays. The metrics are obtained from the unrestricted and restricted VAR(1).

Table 6 reports the coefficients of the VAR(1) and the significance of Granger causality applied to weekly standard deviations of 11 industries. As expected, causality in volatility is more pronounced than causality in the mean. Only 12 out of 132 tests are not significant at the 5% level, and for the $US\to k$ tests only five are not statistically significant at the 5% level. Hence, the causal relationship from the US is very strong, with most statistics showing a *p* value lower than 1%. Thus, the US volatility is an important leading indicator of the industry turbulence in other countries. However, the causal relationship is less asymmetric than in the mean. For instance, for France and the UK, there is causality in both directions for all industries with a significance level of 1%. Moreover, Canadian and Japanese volatilities cause the US volatilities, except for real estate (RE) and utilities (UT). In Germany, there is causality from and to the US, except for real estate (RE). Once again, China presents the lowest number of significant causal relationships (in both directions). Nevertheless, the volatility in the US Granger causes seven out of 11 Chinese industries.

**Table 6:** Coefficients of the VAR(1) and significance of Granger causality in volatility

	BM	CD	CS	EN	FI	HC	IN	RE	TEC	TEL	UT
$US\to CN$	0.235*	0.320*	0.271*	0.219*	0.274*	0.108*	0.320*	0.060*	0.262*	0.200*	0.260*
$CN\to US$	0.190*	0.116*	0.084*	0.189*	0.134*	**0.021**	0.121*	0.038	0.095*	0.140*	0.123*
$US\to FR$	0.288*	0.335*	0.320*	0.261*	0.290*	0.276*	0.301*	0.119*	0.291*	0.098*	0.138*
$FR\to US$	0.152*	0.125*	0.076*	0.162*	0.123*	0.113*	0.106*	0.101*	0.129*	0.102*	0.126*
$US\to GE$	0.269*	0.435*	0.276*	**0.037**	0.244*	0.207*	0.259*	− 0.009	0.093*	0.322*	0.182*
$GE\to US$	0.151*	0.033*	0.044*	0.112*	0.122*	0.120*	0.150*	− 0.015	0.106*	0.135*	0.095*
$US\to JP$	0.187*	0.199*	0.216*	0.189*	0.154*	0.181*	0.207*	0.092*	0.185*	0.174*	0.073*
$JP\to US$	0.091*	0.112*	0.078*	0.080*	0.062*	0.075*	0.102*	0.052*	0.145*	0.033*	0.006*
$US\to CH$	0.135*	0.112*	0.090	0.116*	0.124*	0.004	0.085*	− 0.017	− 0.020*	0.032	0.196*
$CH\to US$	0.059*	0.028*	0.006	0.067*	0.065*	− 0.002	0.052*	− 0.007	− 0.016	0.053*	0.058*
$US\to UK$	0.249*	0.218*	0.214*	0.271*	0.310*	0.243*	0.256*	0.221*	0.230*	0.257*	0.201*
$UK\to US$	0.139*	0.135*	0.110*	0.222*	0.150*	0.119*	0.074*	0.236*	0.102*	0.138*	0.123*

This table presents the coefficients of a bivariate VAR(1) and the significance of the Granger causality tests applied to weekly volatilities. Data cover different periods, all ending on 17/05/2021 (see Table 1). The tests are conducted pairwise between the US and other six countries (Canada (CN), France (FR), Germany (GE), Japan (JP), China (CH), and the UK) for 11 industries (Basic Materials (BM), Consumer Discretionary (CD), Consumer Staples (CS), Energy (EN), Financials (FI), Health Care (HC), Industrials (IN), Real Estate (RE), Technology (TEC), Telecommunications (TEL), and Utilities (UT)). $US\to k$ indicates Granger causality from the US to the returns of country $k$, and $k\to US$ indicates Granger causality from country $k$ to the US, for each industry. Numbers in bold indicate the rejection of the null hypothesis of no Granger causality at the 5% level. One asterisk, “*”, denotes significance at the 1% level

The pairwise relations in volatility between US and non-US countries are further analyzed using the Geweke feedback measures reported in Table 7. As expected, the feedback measures applied to volatilities were more significant than the corresponding figures for returns (see Table 4). However, once again, the percentage of lagged feedback in the volatility from the US to the other six countries is, in general, higher than the feedback in the opposite direction. The only exception is China, where the lagged feedback is higher for most industries than the feedback from the US, with an average value of 21% in relative terms (average value across Chinese industries), which suggests that volatility flows mainly from the Chinese to the US market. This situation also appears in some industries in France, Germany, and the UK. Nevertheless, the results show that lagged feedback in volatility is globally asymmetric and predominantly dominated by the US.

**Table 7:** Geweke feedback measures in volatility

	BM	CD	CS	EN	FI	HC	IN	RE	TEC	TEL	UT
$${F}_{US\to k}$$											
CN	0.039^*^	0.090^*^	0.068^*^	0.031^*^	0.086^*^	0.003^*^	0.064^*^	0.010^*^	0.033^*^	0.043^*^	0.068^*^
	8.8%	27.4%	26.9%	5.3%	17.1%	3.6%	14.4%	13.5%	11.7%	24.2%	27.9%
FR	0.093^*^	0.075^*^	0.054^*^	0.063^*^	0.084^*^	0.059^*^	0.079^*^	0.052^*^	0.064^*^	0.007^*^	0.014^*^
	36.4%	35.3%	39.1%	24.3%	31.0%	33.5%	33.2%	46.4%	37.4%	7.6%	10.0%
GE	0.084^*^	0.060^*^	0.029^*^	0.002	0.079^*^	0.047^*^	0.060^*^	0.000	0.007^*^	0.058^*^	0.038^*^
	28.6%	49.1%	38.9%	2.0%	29.3%	23.5%	21.8%	2.9%	6.8%	30.3%	30.6%
JP	0.042^*^	0.038^*^	0.029^*^	0.034^*^	0.030^*^	0.030^*^	0.041^*^	0.014^*^	0.041^*^	0.013^*^	0.004^*^
	26.8%	26.0%	24.9%	37.8%	28.6%	23.3%	26.1%	33.6%	31.7%	35.4%	9.0%
CH	0.011^*^	0.004^*^	0.001	0.011^*^	0.016^*^	0.000	0.004^*^	0.000	0.002	0.001	0.017^*^
	14.9%	14.7%	14.1%	21.7%	31.4%	0.1%	12.5%	7.7%	13.8%	5.0%	27.5%
UK	0.037^*^	0.042^*^	0.035^*^	0.067^*^	0.101^*^	0.046^*^	0.062^*^	0.084^*^	0.035^*^	0.040^*^	0.039^*^
	12.6%	22.1%	17.9%	18.8%	31.9%	22.7%	31.1%	41.0%	29.6%	21.6%	28.0%
$${F}_{k\to US}$$											
CN	0.034^*^	0.012^*^	0.007^*^	0.026^*^	0.010^*^	0.003^*^	0.014^*^	0.001	0.017^*^	0.022^*^	0.017^*^
	7.7%	3.6%	2.6%	4.4%	2.0%	2.8%	3.3%	1.9%	5.9%	12.8%	7.0%
FR	0.022^*^	0.025^*^	0.012^*^	0.027^*^	0.018^*^	0.018^*^	0.014^*^	0.005^*^	0.026^*^	0.035^*^	0.056^*^
	8.5%	11.7%	9.0%	10.2%	6.6%	10.1%	6.0%	4.3%	15.2%	37.5%	40.7%
GE	0.018^*^	0.004^*^	0.007^*^	0.028^*^	0.013^*^	0.013^*^	0.024^*^	0.000	0.026^*^	0.037^*^	0.013^*^
	6.2%	3.4%	9.0%	33.5%	4.9%	6.5%	8.8%	4.5%	25.0%	19.4%	10.6%
JP	0.009^*^	0.015^*^	0.012^*^	0.009^*^	0.006^*^	0.007^*^	0.014^*^	0.004^*^	0.023^*^	0.004^*^	0.000
	5.9%	10.5%	10.0%	10.2%	5.5%	5.5%	8.7%	8.4%	17.7%	10.9%	0.2%
CH	0.015^*^	0.006^*^	0.001	0.016^*^	0.015^*^	0.000	0.013^*^	0.000	0.000	0.010^*^	0.022^*^
	18.8%	20.9%	5.0%	31.2%	29.4%	0.1%	37.0%	10.5%	2.0%	43.2%	36.8%
UK	0.033^*^	0.024^*^	0.016^*^	0.043^*^	0.023^*^	0.018^*^	0.007^*^	0.049^*^	0.021^*^	0.041^*^	0.028^*^
	11.3%	12.3%	8.3%	12.0%	7.2%	9.2%	3.3%	23.8%	17.5%	22.1%	19.9%
$${F}_{US\leftrightarrow k}$$											
CN	0.371^*^	0.228^*^	0.179^*^	0.521^*^	0.407^*^	0.084^*^	0.365^*^	0.063^*^	0.234^*^	0.111^*^	0.158^*^
	83.5%	69.0%	70.5%	90.2%	80.9%	93.6%	82.3%	84.6%	82.5%	63.0%	65.2%
FR	0.141^*^	0.112^*^	0.072^*^	0.171^*^	0.169^*^	0.099^*^	0.144^*^	0.056^*^	0.081^*^	0.051^*^	0.067^*^
	55.1%	53.0%	51.9%	65.5%	62.4%	56.4%	60.9%	49.3%	47.4%	54.9%	49.3%
GE	0.192^*^	0.058^*^	0.038^*^	0.055^*^	0.179^*^	0.140^*^	0.190^*^	0.007^*^	0.071^*^	0.097^*^	0.072^*^
	65.1%	47.5%	52.1%	64.4%	65.9%	70.0%	69.4%	92.6%	68.2%	50.4%	58.8%
JP	0.105^*^	0.093^*^	0.077^*^	0.047^*^	0.070^*^	0.093^*^	0.103^*^	0.025^*^	0.065^*^	0.020^*^	0.039^*^
	67.2%	63.5%	65.1%	52.0%	65.9%	71.2%	65.2%	58.0%	50.6%	53.7%	90.8%
CH	0.051^*^	0.019^*^	0.008^*^	0.024^*^	0.020^*^	0.017^*^	0.018^*^	0.002	0.011^*^	0.012^*^	0.021^*^
	66.3%	64.4%	80.9%	47.2%	39.3%	99.9%	50.5%	81.8%	84.3%	51.8%	35.7%
UK	0.223^*^	0.126^*^	0.145^*^	0.245^*^	0.192^*^	0.137^*^	0.130^*^	0.072^*^	0.062^*^	0.104^*^	0.073^*^
	76.1%	65.6%	73.8%	69.2%	60.9%	68.2%	65.6%	35.2%	52.9%	56.3%	52.1%
$${F}_{US,k}$$											
CN	0.445^*^	0.330^*^	0.254^*^	0.577^*^	0.503^*^	0.090^*^	0.443^*^	0.075^*^	0.283^*^	0.176^*^	0.242^*^
FR	0.257^*^	0.211^*^	0.138^*^	0.261^*^	0.272^*^	0.176^*^	0.237^*^	0.113^*^	0.170^*^	0.093^*^	0.137^*^
GE	0.295^*^	0.122^*^	0.073^*^	0.085^*^	0.271^*^	0.200^*^	0.274^*^	0.007^*^	0.104^*^	0.192^*^	0.123^*^
JP	0.156^*^	0.147^*^	0.118^*^	0.091^*^	0.106^*^	0.130^*^	0.158^*^	0.043^*^	0.128^*^	0.037^*^	0.043^*^
CH	0.077^*^	0.029^*^	0.010^*^	0.050^*^	0.050^*^	0.017^*^	0.036^*^	0.002	0.013^*^	0.022^*^	0.060^*^
UK	0.294^*^	0.192^*^	0.196^*^	0.354^*^	0.316^*^	0.201^*^	0.199^*^	0.206^*^	0.117^*^	0.185^*^	0.141^*^

This table presents the Geweke feedback measures resulting from a bivariate VAR(1) applied to weekly volatilities. Data cover different periods, all ending on 17/05/2021 (see Table 1). The tests are conducted pairwise between the US and other 6 countries (Canada (CN), France (FR), Germany (GE), Japan (JP), China (CH), and the UK) for 11 industries (Basic Materials (BM), Consumer Discretionary (CD), Consumer Staples (CS), Energy (EN), Financials (FI), Health Care (HC), Industrials (IN), Real Estate (RE), Technology (TEC), Telecommunications (TEL) and Utilities (UT)). ${F}_{US\to k}$ is the measure of lagged feedback from the US to country $k$, ${F}_{k\to US}$ is the measure of lagged feedback from country $k$ to the US, ${F}_{US\leftrightarrow k}$ is the measure of contemporaneous feedback$,$ and ${F}_{US,k}$ is the measure of total feedback. Numbers in bold indicate the rejection of the null of no feedback at the 5% level. One asterisk, “*”, denotes significance at the 1% level. Numbers in italic represent the weight of the lagged and contemporaneous feedbacks to the total feedback

Contemporaneous feedback is the main contributor to the total feedback, with percentages ranging from 35.2% for real estate (RE) in the UK to 99.9% for health care (HC) in China, with a global average value of 65%. These results suggest that most markets are integrated and that, on average, 65% of volatility is communicated within one week.

At the industry level, the basic materials (BM), energy (EN), and financial (FI) industries report high levels of feedback transmission. This is justified by these industries containing the largest companies in the world, where volatility shocks spread more rapidly (World Bank 2021). The results of Buncic and Gisler (2016) support our findings. These authors show that the daily realized volatility data for the US play an overwhelmingly strong role in 17 international equity markets for up to 22 days. However, one should note that the linkage between volatility across countries may be longer lived, as suggested by Bollerslev et al. (2014). The authors define a capitalization-weighted global variance risk premium, which is used to uncover a stronger predictability with a systematic peak in the degree of predictability around the 4-month horizon and almost identical cross-country patterns using panel regressions for France, Germany, Japan, Switzerland, the Netherlands, Belgium, the UK, and the US.

### Causality and feedback during expansions and recessions

This subsection analyzes causality and feedback during the expansion and recession periods in the US, which were identified using the NBER business cycle classification. Table 8 reports the coefficients of VAR(1) and the significance of the Granger causality in the mean during expansions and recessions.

**Table 8:** Coefficients of the VAR(1) and significance of Granger causality in the mean during expansions and recessions

	BM	CD	CS	EN	FI	HC	IN	RE	TEC	TEL	UT
*Expansions*											
$US\to CN$	0.066	0.102*	0.049	0.020	0.021	0.045	− 0.011	0.037	− 0.010	**0.055**	0.077
$CN\to US$	− 0.012	− 0.025	0.000	**0.049**	− 0.010	**− 0.026**	0.019	0.008	0.031	**− 0.055**	0.024
$US\to FR$	0.048	0.022	0.017	0.093*	− 0.037	0.008	− 0.019	− 0.008	0.005	− 0.006	0.048
$FR\to US$	0.027	− 0.001	− 0.008	0.021	− 0.024	0.005	0.005	0.005	0.051*	− 0.014	− 0.072
$US\to GE$	0.067*	0.035	**0.068**	− 0.002	− 0.002	0.048	0.033	− 0.002	0.020	− 0.023	0.026*
$GE\to US$	0.025	− 0.021	0.013	0.016	− 0.035	0.022	− 0.019	− 0.013	0.071*	0.018	− 0.012
$US\to JP$	0.081*	0.092*	**0.067**	0.131*	**0.066**	0.114*	0.134*	0.064*	0.145*	**0.093**	0.112*
$JP\to US$	− 0.032	− 0.063*	**− 0.033**	0.041*	− 0.010	− 0.026	− 0.017	0.005	− 0.002	− 0.020	0.012
$US\to CH$	0.150*	0.008	0.020	0.109*	0.051	0.034	0.034	− 0.013	0.005	0.042	0.029*
$CH\to US$	0.008	− 0.008	− 0.007	**0.036**	− 0.029	0.023	− 0.004	0.008	0.055	− 0.005	− 0.020
$US\to UK$	0.081*	0.071*	**0.064**	0.115*	0.035	0.046	0.044	0.041	0.113*	0.022	**0.022**
$UK\to US$	0.019	− 0.005	0.024	0.074*	− 0.017	0.016	0.003	0.030	0.020	− 0.018	0.000
*Recessions*											
$US\to CN$	**0.223**	0.298*	0.110	**0.216**	0.131	0.265*	**0.222**	0.066	0.012	**0.121**	**0.150**
$CN\to US$	− 0.113	− 0.032	0.196*	− 0.035	0.069	0.031	− 0.026	− 0.084	**0.112**	− 0.068	0.078
$US\to FR$	0.195*	0.129	0.032	**0.175**	0.258*	0.028	0.140	0.129*	0.102	0.002	0.233*
$FR\to US$	0.021	**0.119**	0.145*	0.006	− 0.066	**0.108**	0.029	0.012	0.050	− 0.041	0.059
$US\to GE$	0.196*	0.307*	0.052	0.196*	0.191*	0.146*	0.125	0.044	0.002	**0.193**	**0.152**
$GE\to US$	− 0.061	0.003	0.089	− 0.034	− 0.003	0.065	0.003	0.029	0.039	− 0.007	0.031
$US\to JP$	0.215*	0.211*	**0.161**	0.183*	0.200*	0.189*	0.219*	0.153*	0.221*	0.195*	**0.098**
$JP\to US$	0.045	− 0.015	0.038	− 0.055	**− 0.158**	0.028	− 0.011	− 0.029	− 0.033	− 0.041	0.014
$US\to CH$	0.295*	0.092	0.074*	0.144	0.069	0.134	0.110	0.074	0.006	**0.130**	− 0.008
$CH\to US$	**− 0.090**	− 0.019	0.032	− 0.017	− 0.169*	0.013	**− 0.090**	− 0.169*	− 0.033	− 0.070	0.058
$US\to UK$	0.290*	0.230*	**0.154**	**0.207**	**0.184**	**0.157**	**0.204**	0.229*	0.139	**0.185**	0.074
$UK\to US$	− 0.029	− 0.001	0.149*	0.061	− 0.066	0.124*	0.006	− 0.022	− 0.028	− 0.077	0.030

This table presents the coefficients of a bivariate VAR(1) and the significance of the Granger causality tests applied to weekly returns using data in periods identified as expansions or recessions in the US according to the NBER business cycle classification (https://www.nber.org/research/data/us-business-cycle-expansions-and-contractions). The tests are conducted pairwise between the US and other 6 countries (Canada (CN), France (FR), Germany (GE), Japan (JP), China (CH), and the UK) for 11 industries (Basic Materials (BM), Consumer Discretionary (CD), Consumer Staples (CS), Energy (EN), Financials (FI), Health Care (HC), Industrials (IN), Real Estate (RE), Technology (TEC), Telecommunications (TEL) and Utilities (UT)). $US\to k$ indicates Granger causality from the US to the returns of country $k$, and $k\to US$ indicates Granger causality from country $k$ to the US, for each industry. Numbers in bold indicate the rejection of the null hypothesis of no Granger causality at the 5% level. One asterisk, “*”, denotes significance at the 1% level

The transmission of information mainly flows from the US to other countries during expansion and recession periods. It is visibly less pronounced during expansion periods, although the US continues to dominate other countries during these periods. The decrease in causality extends across all the countries. For instance, the US returns only Granger cause Canadian returns in less than half of the industries, while for the full sample, Granger causes Canadian returns in ten out of 11 industries. A similar situation occurs for Germany and the UK, where US returns only Granger cause three and six industries, respectively. The exception to this pattern is Japan, for which the US returns Granger cause most industry returns in expansion and recession periods. The differences in the causal relationships between countries during expansions and recessions are notorious, implying that more information is transmitted during recession periods. Other papers reached similar or compatible results (see, for instance, Henkel et al. 2011; Ji et al. 2020; Salisu et al. 2022).

Tables 9 and 10 present the feedback measures during the expansion and recession periods, respectively. The total feedback is lower during the expansion periods; on average, it is 0.166 and 0.316 during expansions and recessions, respectively. During recessions, the average unidirectional feedback was also higher than that during expansions (5.57% vs. 2.02%, respectively). However, there is a different pattern for the contemporaneous feedback. Despite remaining the dominant contributor to total feedback, we observe that this feedback is higher during expansions than during recessions in relative terms. The average relative contemporaneous feedback during the expansion was 96%: it was 89% during the recessions and 94% during the full sample period. This indicates that during an expansionary period, there is a 2% increase in the transmission of information communicated between markets within one week relative to a recession period.

**Table 9:** Geweke feedback measures in the mean during expansions

	BM	CD	CS	EN	FI	HC	IN	RE	TEC	TEL	UT
$${F}_{US\to k}$$											
CN	0.002	0.007^*^	0.002	0.000	0.000	0.000	0.000	0.002	0.000	**0.002**	0.005^*^
	*0.2%*	*1.9%*	*0.8%*	*0.0%*	*0.1%*	*0.3%*	*0.0%*	*1.0%*	*0.0%*	*2.0%*	*3.9%*
FR	**0.002**	0.000	0.000	0.004^*^	0.001	0.000	0.000	0.000	0.000	0.000	0.001
	*0.5%*	*0.1%*	*0.1%*	*1.3%*	*0.2%*	*0.0%*	*0.1%*	*0.2%*	*0.0%*	*0.0%*	*1.1%*
GE	0.004^*^	0.000	0.002	0.000	0.000	**0.002**	0.001	0.000	0.000	0.000	0.000
	*1.2%*	*0.3%*	*3.6%*	*0.0%*	*0.0%*	*1.0%*	*0.2%*	*0.0%*	*0.1%*	*0.2%*	*0.9%*
JP	0.005^*^	0.006^*^	**0.002**	0.008^*^	**0.002**	0.009^*^	0.011^*^	**0.002**	0.014^*^	**0.003**	**0.004**
	*5.2%*	*5.6%*	*4.1%*	*9.7%*	*3.5%*	*14.8%*	*8.6%*	*9.7%*	*9.6%*	*8.6%*	*18.0%*
CH	0.010^*^	0.000	0.000	0.006^*^	**0.001**	0.001	0.000	0.000	0.000	**0.002**	0.000
	*11.4%*	*0.1%*	*1.0%*	*7.7%*	*4.3%*	*5.1%*	*1.6%*	*1.5%*	*0.7%*	*10.4%*	*1.3%*
UK	0.003^*^	0.003^*^	0.002^*^	**0.006**	0.001	0.001	0.001	0.001	0.007^*^	0.000	0.000
	*0.9%*	*1.9%*	*1.3%*	*1.3%*	*0.2%*	*0.5%*	*0.5%*	*2.5%*	*4.6%*	*0.2%*	*0.5%*
$${F}_{k\to US}$$											
CN	0.000	0.000	0.000	**0.002**	0.000	**0.002**	0.000	0.000	0.001	**0.003**	0.001
	*0.0%*	*0.1%*	*0.0%*	*0.3%*	*0.0%*	*1.2%*	*0.1%*	*0.0%*	*0.3%*	*2.3%*	*0.4%*
FR	0.001	0.000	0.000	0.001	0.001	0.000	0.000	0.000	0.004	0.000	0.008^*^
	*0.2%*	*0.0%*	*0.1%*	*0.2%*	*0.3%*	*0.0%*	*0.0%*	*0.0%*	*1.9%*	*0.3%*	*7.2%*
GE	0.000	0.001	0.000	0.000	**0.001**	0.000	0.000	0.000	0.006^*^	0.001	0.000
	*0.2%*	*0.5%*	*0.6%*	*0.5%*	*0.5%*	*0.3%*	*0.1%*	*0.4%*	*3.6%*	*0.6%*	*0.5%*
JP	0.001	0.004^*^	**0.002**	0.003^*^	0.000	0.001	0.000	0.000	0.000	0.001	0.000
	*1.2%*	*3.8%*	*3.8%*	*4.3%*	*0.3%*	*1.6%*	*0.3%*	*0.2%*	*0.0%*	*3.7%*	*2.1%*
CH	0.000	0.000	0.000	**0.003**	**0.002**	0.001	0.000	0.000	0.001	0.000	**0.001**
	*0.1%*	*0.8%*	*4.3%*	*3.5%*	*6.5%*	*6.4%*	*0.2%*	*3.8%*	*6.4%*	*0.2%*	*7.2%*
UK	0.000	0.000	0.001	0.005^*^	0.000	0.000	0.000	0.001	0.001	0.001	0.000
	*0.1%*	*0.0%*	*0.5%*	*1.0%*	*0.1%*	*0.1%*	*0.0%*	*2.0%*	*0.4%*	*0.4%*	*0.0%*
$${F}_{US\leftrightarrow k}$$											
CN	0.680^*^	0.374^*^	0.192^*^	0.577^*^	0.494^*^	0.167^*^	0.502^*^	0.176^*^	0.398^*^	0.118^*^	0.126^*^
	*99.7%*	*98.0%*	*99.2%*	*99.6%*	*99.9%*	*98.5%*	*99.9%*	*99.0%*	*99.7%*	*95.7%*	*95.7%*
FR	0.279^*^	0.213^*^	0.122^*^	0.279^*^	0.268^*^	0.180^*^	0.228^*^	0.049^*^	0.202^*^	0.101^*^	0.102^*^
	*99.2%*	*99.9%*	*99.8%*	*98.5%*	*99.5%*	*100%*	*99.9%*	*99.8%*	*98.1%*	*99.7%*	*91.7%*
GE	0.289^*^	0.150^*^	0.052^*^	0.062^*^	0.248^*^	0.168^*^	0.261^*^	0.035^*^	0.165^*^	0.104^*^	0.045^*^
	*98.6%*	*99.1%*	*95.8%*	*99.5%*	*99.5%*	*98.7%*	*99.6%*	*99.6%*	*96.3%*	*99.2%*	*98.7%*
JP	0.089^*^	0.103^*^	0.049^*^	0.067^*^	0.061^*^	0.049^*^	0.121^*^	0.022^*^	0.133^*^	0.028^*^	0.017^*^
	*93.7%*	*90.6%*	*92.1%*	*86.0%*	*96.2%*	*83.6%*	*91.1%*	*90.2%*	*90.3%*	*87.7%*	*79.9%*
CH	0.077^*^	0.024^*^	0.007^*^	0.064^*^	0.024^*^	0.012^*^	0.027^*^	0.004^*^	0.012^*^	0.014^*^	0.017^*^
	*88.5%*	*99.2%*	*94.8%*	*88.8%*	*89.3%*	*88.4%*	*98.2%*	*94.8%*	*92.9%*	*89.4%*	*91.5%*
UK	0.349^*^	0.167^*^	0.169^*^	0.460^*^	0.313^*^	0.231^*^	0.206^*^	0.051^*^	0.139^*^	0.138^*^	0.069^*^
	*99.0%*	*98.1%*	*98.3%*	*97.6%*	*99.7%*	*99.4%*	*99.5%*	*95.5%*	*95.0%*	*99.4%*	*99.5%*
$${F}_{US,k}$$											
CN	0.682^*^	0.382^*^	0.194^*^	0.580^*^	0.495^*^	0.170^*^	0.503^*^	0.178^*^	0.399^*^	0.124^*^	0.131^*^
FR	0.281^*^	0.213^*^	0.122^*^	0.284^*^	0.270^*^	0.181^*^	0.228^*^	0.049^*^	0.206^*^	0.101^*^	0.111^*^
GE	0.293^*^	0.152^*^	0.054^*^	0.062^*^	0.249^*^	0.170^*^	0.262^*^	0.035^*^	0.172^*^	0.105^*^	0.046^*^
JP	0.095^*^	0.114^*^	0.053^*^	0.078^*^	0.063^*^	0.058^*^	0.132^*^	0.025^*^	0.147^*^	0.032^*^	0.022^*^
CH	0.087^*^	0.024^*^	0.007^*^	0.072^*^	0.027^*^	0.014^*^	0.028^*^	0.004^*^	0.013^*^	0.016^*^	0.019^*^
UK	0.352^*^	0.170^*^	0.171^*^	0.472^*^	0.314^*^	0.232^*^	0.207^*^	0.053^*^	0.146^*^	0.139^*^	0.070^*^

This table presents the Geweke feedback measures resulting from a bivariate VAR(1) applied to weekly returns using data in periods identified as expansions in the US according to the NBER business cycle classification (https://www.nber.org/research/data/us-business-cycle-expansions-and-contractions). The tests are conducted pairwise between the US and other 6 countries (Canada (CN), France (FR), Germany (GE), Japan (JP), China (CH)), and the UK) for 11 industries (Basic Materials (BM), Consumer Discretionary (CD), Consumer Staples (CS), Energy (EN), Financials (FI), Health Care (HC), Industrials (IN), Real Estate (RE), Technology (TEC), Telecommunications (TEL) and Utilities (UT)). ${F}_{US\to k}$ is the measure of lagged feedback from the US to country$k$, ${F}_{k\to US}$ is the measure of lagged feedback from country $k$ to the US, ${F}_{US\leftrightarrow k}$ is the measure of contemporaneous feedback$,$ and ${F}_{US,k}$ is the measure of total feedback. Numbers in bold indicate the rejection of the null of no feedback at the 5% level. One asterisk, “*”, denotes significance at the 1% level. Numbers in italic represent the weight of the lagged and contemporaneous feedbacks to the total feedback

**Table 10:** Geweke feedback measures in the mean during recessions

	BM	CD	CS	EN	FI	HC	IN	RE	TEC	TEL	UT
$${F}_{US\to k}$$											
CN	**0.015**	0.039^*^	**0.009**	**0.014**	**0.010**	0.021^*^	**0.015**	**0.007**	**0.000**	**0.012**	**0.012**
	*1.8%*	*7.7%*	*3.8%*	*1.4%*	*1.3%*	*10.9%*	*1.9%*	*2.3%*	*0.0%*	*5.9%*	*2.7%*
FR	0.030^*^	**0.008**	0.001	**0.016**	0.030^*^	0.000	0.008	0.026^*^	0.004	0.000	0.024
	*6.6%*	*2.0%*	*0.3%*	*2.7%*	*5.0%*	*0.2%*	*1.6%*	*11.9%*	*1.1%*	*0.0%*	*9.8%*
GE	0.028^*^	0.040^*^	0.002	0.030^*^	0.022^*^	0.020^*^	**0.007**	0.004	0.000	**0.017**	**0.016**
	*4.6%*	*17.1%*	*2.9%*	*10.3%*	*4.0%*	*12.1%*	*1.4%*	*2.5%*	*0.0%*	*11.3%*	*7.1%*
JP	0.050^*^	0.038^*^	**0.013**	0.028^*^	0.043^*^	0.035^*^	0.038^*^	0.033^*^	0.039^*^	0.019^*^	0.010
	*22.4%*	*15.6%*	*10.6%*	*13.1%*	*16.0%*	*25.6%*	*16.8%*	*22.3%*	*20.7%*	*37.6%*	*31.2%*
CH	0.039^*^	0.003	0.002	0.022^*^	0.005	0.006	0.007	0.007	0.000	**0.011**	0.000
	*17.3%*	*4.8%*	*10.9%*	*15.8%*	*2.1%*	*12.4%*	*6.6%*	*11.6%*	*0.2%*	*10.7%*	*0.0%*
UK	0.023^*^	0.019^*^	**0.011**	**0.016**	**0.014**	**0.011**	**0.016**	0.040^*^	**0.010**	**0.017**	0.006
	*3.3%*	*4.3%*	*3.8%*	*2.1%*	*2.3%*	*3.9%*	*2.8%*	*16.3%*	*2.6%*	*6.4%*	*2.9%*
$${F}_{k\to US}$$											
CN	**0.009**	0.001	0.036^*^	0.001	0.002	0.002	0.000	0.002	**0.017**	0.004	0.005
	*1.1%*	*0.2%*	*15.2%*	*0.1%*	*0.2%*	*1.2%*	*0.1%*	*0.7%*	*4.1%*	*1.9%*	*1.2%*
FR	0.000	**0.015**	0.026^*^	0.000	0.003	**0.012**	0.001	0.000	0.003	0.002	0.005
	*0.1%*	*4.1%*	*12.2%*	*0.0%*	*0.5%*	*5.6%*	*0.1%*	*0.0%*	*0.7%*	*1.0%*	*2.1%*
GE	0.002	0.000	**0.009**	0.001	0.000	0.003	0.000	0.000	0.001	0.000	0.001
	*0.3%*	*0.0%*	*11.7%*	*0.3%*	*0.0%*	*2.1%*	*0.0%*	*0.1%*	*0.2%*	*0.1%*	*0.4%*
JP	0.001	0.000	0.002	0.003	**0.015**	0.001	0.000	0.000	0.001	0.003	0.000
	*0.6%*	*0.1%*	*1.9%*	*1.2%*	*5.6%*	*0.5%*	*0.1%*	*0.3%*	*0.6%*	*6.1%*	*0.5%*
CH	**0.013**	0.001	0.003	0.000	0.021^*^	0.000	**0.012**	0.022^*^	0.000	0.007	0.007
	*5.7%*	*1.3%*	*15.8%*	*0.1%*	*9.6%*	*0.9%*	*11.1%*	*35.7%*	*0.4%*	*6.7%*	*11.9%*
UK	0.001	0.000	0.031^*^	0.002	0.003	0.020^*^	0.000	0.000	0.001	0.007	0.001
	*0.1%*	*0.0%*	*10.9%*	*0.3%*	*0.5%*	*6.9%*	*0.0%*	*0.2%*	*0.2%*	*2.8%*	*0.2%*
$${F}_{US\leftrightarrow k}$$											
CN	0.800^*^	0.474^*^	0.190^*^	0.974^*^	0.774^*^	0.170^*^	0.772^*^	0.316^*^	0.385^*^	0.190^*^	0.423^*^
	*97.2%*	*92.2%*	*81.0%*	*98.5%*	*98.5%*	*87.9%*	*98.0%*	*97.0%*	*95.9%*	*92.2%*	*96.2%*
FR	0.432^*^	0.353^*^	0.188^*^	0.554^*^	0.565^*^	0.210^*^	0.500^*^	0.194^*^	0.403^*^	0.154^*^	0.214^*^
	*93.4%*	*93.9%*	*87.6%*	*97.3%*	*94.4%*	*94.2%*	*98.3%*	*88.1%*	*98.3%*	*99.0%*	*88.0%*
GE	0.567^*^	0.194^*^	0.064^*^	0.256^*^	0.523^*^	0.143^*^	0.524^*^	0.171^*^	0.479^*^	0.131^*^	0.201^*^
	*95.1%*	*82.9%*	*85.4%*	*89.4%*	*96.0%*	*85.8%*	*98.6%*	*97.3%*	*99.8%*	*88.6%*	*92.4%*
JP	0.174^*^	0.207^*^	0.109^*^	0.179^*^	0.213^*^	0.102^*^	0.188^*^	0.115^*^	0.147^*^	0.029^*^	0.023^*^
	*77.0%*	*84.3%*	*87.5%*	*85.6%*	*78.4%*	*73.9%*	*83.2%*	*77.4%*	*78.7%*	*56.3%*	*68.3%*
CH	0.174^*^	0.062^*^	0.013^*^	0.119^*^	0.189^*^	0.043^*^	0.088^*^	0.032^*^	0.063^*^	0.083^*^	0.054^*^
	*77.0%*	*93.9%*	*73.3%*	*84.1%*	*88.3%*	*86.8%*	*82.3%*	*52.7%*	*99.4%*	*82.6%*	*88.1%*
UK	0.670^*^	0.413^*^	0.242^*^	0.735^*^	0.594^*^	0.259^*^	0.562^*^	0.208^*^	0.363^*^	0.241^*^	0.212^*^
	*96.5%*	*95.7%*	*85.3%*	*97.6%*	*97.2%*	*89.2%*	*97.2%*	*83.6%*	*97.2%*	*90.8%*	*96.8%*
$${F}_{US,k}$$											
CN	0.824^*^	0.515^*^	0.235^*^	0.989^*^	0.786^*^	0.194^*^	0.788^*^	0.326^*^	0.402^*^	0.207^*^	0.440^*^
FR	0.463^*^	0.376^*^	0.215^*^	0.569^*^	0.599^*^	0.223^*^	0.509^*^	0.220^*^	0.410^*^	0.156^*^	0.244^*^
GE	0.596^*^	0.234^*^	0.075^*^	0.287^*^	0.544^*^	0.167^*^	0.531^*^	0.176^*^	0.480^*^	0.147^*^	0.217^*^
JP	0.225^*^	0.245^*^	0.124^*^	0.210^*^	0.271^*^	0.138^*^	0.227^*^	0.149^*^	0.187^*^	0.051^*^	0.033^*^
CH	0.226^*^	0.066^*^	0.018^*^	0.142^*^	0.214^*^	0.050^*^	0.107^*^	0.060^*^	0.063^*^	0.100^*^	0.061^*^
UK	0.694^*^	0.431^*^	0.283^*^	0.753^*^	0.611^*^	0.290^*^	0.578^*^	0.249^*^	0.374^*^	0.265^*^	0.218^*^

This table presents the Geweke feedback measures resulting from a bivariate VAR(1) applied to weekly returns using data in periods identified as recessions in the US according to the NBER business cycle classification (https://www.nber.org/research/data/us-business-cycle-expansions-and-contractions). The tests are conducted pairwise between the US and other 6 countries (Canada (CN), France (FR), Germany (GE), Japan (JP), China (CH), and the UK) for 11 industries (Basic Materials (BM), Consumer Discretionary (CD), Consumer Staples (CS), Energy (EN), Financials (FI), Health Care (HC), Industrials (IN), Real Estate (RE), Technology (TEC), Telecommunications (TEL) and Utilities (UT)). ${F}_{US\to k}$ is the measure of lagged feedback from the US to country$k$, ${F}_{k\to US}$ is the measure of lagged feedback from country $k$ to the US, ${F}_{US\leftrightarrow k}$ is the measure of contemporaneous feedback$,$ and ${F}_{US,k}$ is the measure of total feedback. Numbers in bold indicate the rejection of the null of no feedback at the 5% level. One asterisk, “*”, denotes significance at the 1% level. Numbers in italic represent the weight of the lagged and contemporaneous feedbacks to the total feedback

In conclusion, we observe that during a recession, linear dependence increases, but the time that countries take to adjust to new information is higher than during an expansion, suggesting that investors react with a larger delay. Arguably, during a recession, levels of uncertainty tend to be higher and investors’ confidence in information signals tends to decrease.

### Causality in distribution

This subsection examines Granger causality in the distribution using the procedures proposed by Candelon and Tokpavi (2016).

Table 11 presents the *p* values for the tests applied to the left and right tails of the distribution of returns. The tests in the left tail are conducted considering $\alpha$ = 1%, 5%, and 10%, whereas the tests in the right tail consider $\alpha$ = 90%, 95%, and 99%.

**Table 11:** Granger causality in distribution of returns

	BM	CD	CS	EN	FI	HC	IN	RE	TEC	TEL	UT
*Left tail*											
$US\to CN$	0.000^*^	0.794	0.776	0.002^*^	0.373	0.581	0.225	0.563	**0.036**	**0.022**	**0.027**
$CN\to US$	0.121	0.736	0.063	0.972	0.002^*^	**0.032**	0.359	0.001^*^	0.000^*^	0.684	0.172
$US\to FR$	0.855	0.005^*^	0.376	0.005^*^	0.692	0.000^*^	0.000^*^	0.835	0.000^*^	0.547	0.521
$FR\to US$	0.084	0.000^*^	**0.014**	0.365	0.785	0.628	0.006^*^	0.000^*^	**0.047**	0.969	0.653
$US\to GE$	0.000^*^	0.000^*^	0.701	0.542	0.219	0.000^*^	0.000^*^	0.686	0.192	0.525	**0.035**
$GE\to US$	0.000^*^	0.058	0.000^*^	0.329	0.751	0.059	0.744	0.175	0.000^*^	0.000^*^	0.362
$US\to JP$	0.009^*^	0.774	0.180	0.000^*^	0.001^*^	0.000^*^	0.112	**0.011**	0.001^*^	0.709	0.001^*^
$JP\to US$	0.575	0.287	0.149	0.816	0.145	0.676	0.065	0.001^*^	0.000^*^	0.200	0.690
$US\to CH$	0.000^*^	**0.031**	0.461	**0.045**	0.001^*^	**0.027**	0.721	0.704	0.555	0.213	0.902
$CH\to US$	0.256	0.441	0.541	0.167	0.524	**0.021**	0.996	0.169	0.660	0.393	0.811
$US\to UK$	0.059	0.253	0.002^*^	0.077	0.083	0.000^*^	0.266	0.001^*^	**0.045**	0.248	**0.043**
$UK\to US$	0.079	**0.019**	0.000^*^	0.001^*^	0.057	0.182	**0.045**	0.343	0.000^*^	0.777	**0.045**
*Right tail*											
$US\to CN$	0.000*	0.482	0.420	0.367	0.842	0.890	0.003*	0.433	0.554	0.000*	0.543
$CN\to US$	**0.018**	0.435	0.191	0.244	0.470	0.001*	0.230	0.660	0.251	0.282	**0.011**
$US\to FR$	**0.040**	0.988	0.073	0.148	0.979	0.148	0.730	0.265	**0.022**	0.969	0.694
$FR\to US$	0.440	0.134	0.797	0.781	0.850	0.239	0.307	0.434	0.790	0.547	0.693
$US\to GE$	0.255	0.412	0.076	0.960	0.563	0.253	0.327	0.675	0.287	0.080	0.712
$GE\to US$	**0.045**	0.464	0.332	0.477	0.466	0.168	0.077	0.211	0.001*	0.001*	0.731
$US\to JP$	0.619	0.000*	0.139	0.993	0.913	0.000*	0.009*	0.778	0.116	0.659	0.231
$JP\to US$	**0.042**	0.958	0.672	0.147	0.741	**0.016**	0.000*	0.833	0.594	0.312	0.302
$US\to CH$	0.411	0.861	0.278	0.055	0.957	**0.025**	0.145	0.855	0.999	0.000*	0.820
$CH\to US$	0.717	0.159	0.944	**0.049**	0.376	0.135	0.874	0.303	0.110	0.147	0.087
$US\to UK$	0.117	0.280	0.529	**0.018**	0.174	0.086	0.761	0.366	0.814	0.660	0.345
$UK\to US$	0.335	0.777	0.845	0.395	0.795	0.618	0.217	0.750	0.159	0.996	0.346

This table presents the p-values of the Granger causality in distribution of weekly returns. Data cover different periods, all ending on 17/05/2021 (see Table 1). The tests in the left tail are conducted considering $\alpha$ = 1%, 5% and 10%, while the tests in the right tail consider $\alpha$ = 90%, 95% and 99%. The tests are conducted pairwise between the US and other 6 countries (Canada (CN), France (FR), Germany (GE), Japan (JP), China (CH), and the UK) for 11 industries (Basic Materials (BM), Consumer Discretionary (CD), Consumer Staples (CS), Energy (EN), Financials (FI), Health Care (HC), Industrials (IN), Real Estate (RE), Technology (TEC), Telecommunications (TEL), and Utilities (UT)). $US\to k$ indicates Granger causality from the US to the returns of country $k$, and $k\to US$ indicates Granger causality from country $k$ to the US, for each industry. Numbers in bold indicate the rejection of the null hypothesis of no Granger causality at the 5% level. One asterisk, “*”, denotes significance at the 1% level

For the left tail of the distribution, there is causality from US industries to other countries at the 5% significance level in 32 industries, while causality from other countries to the US occurs in 22 industries. Japan exhibits the highest level of reaction to information coming from the US (seven Japanese industries are Granger caused by the corresponding US industries, at the 1% significance level). For other countries, the number of industries that cause and are caused by US returns is almost always not very different. At the industry level, the technology (TEC) industry presents more significant causalities between the US and other countries.

The results for causality in the right tail reveal less causality. Japan and Canada exhibited the highest number of significant causalities from and to the US.

Table 12 reports the p-values for the left and right tails of the distribution in the volatilities. In the left tail of the distribution, causality is low, mainly flowing from the US to the other countries. On average, the US leads two out of 11 industries for each country. This evidence suggests small information transmission when volatility is low across economies (i.e., in the presence of “calm markets” the lead-lag effect is lessened). However, causality is higher in the right tail of the distribution and mainly flows from the US to other countries. For instance, in France and Germany, eight out of 11 industries react significantly to high volatilities in the US industries. In the case of Canada and the UK, these results also show that other countries do not timely incorporate high volatility shocks that affect US industries. The results for Canada, especially the impact of oil (Energy sector), as previously reported by Salisu et al. (2022), suggest that investors in the Canadian and the US stock markets should consider not only spillovers of tail risks but also the differential impacts of oil market tail risks contingent on the position of these two economies in the oil market.

**Table 12:** Granger causality in distribution of the volatility

	BM	CD	CS	EN	FI	HC	IN	RE	TEC	TEL	UT
*Left tail*											
$US\to CN$	0.220	0.243	0.922	0.000*	0.178	0.609	0.332	0.346	0.223	0.601	**0.020**
$CN\to US$	0.176	0.157	0.061	**0.041**	0.161	0.366	0.098	0.731	0.766	0.621	**0.046**
$US\to FR$	**0.022**	0.912	**0.019**	0.059	0.133	0.562	0.648	0.050	0.323	0.094	0.000*
$FR\to US$	0.794	0.702	**0.034**	0.066	0.633	0.875	0.404	**0.044**	0.773	0.351	0.001*
$US\to GE$	0.117	0.846	**0.010**	–	0.543	0.364	0.083	0.933	0.579	0.001*	0.000*
$GE\to US$	0.794	0.778	0.859	–	0.285	0.174	0.212	0.467	0.078	0.102	0.878
$US\to JP$	0.125	0.533	0.124	0.668	0.119	0.307	0.546	0.233	0.883	0.001*	0.782
$JP\to US$	0.289	0.537	0.007*	0.504	0.598	0.812	0.455	**0.018**	0.495	0.001*	0.469
$US\to CH$	0.579	**0.044**	0.773	0.000*	0.052	–	0.323	0.288	–	–	0.212
$CH\to US$	**0.018**	0.420	0.178	0.415	0.156	–	0.768	0.611	–	**–**	0.057
$US\to UK$	0.209	0.720	0.782	0.581	**0.016**	**0.036**	0.172	0.160	0.428	0.352	**0.047**
$UK\to US$	**0.036**	0.314	0.747	0.169	0.003*	**0.024**	0.085	0.117	0.490	**0.018**	0.108
*Right tail*											
$US\to CN$	0.000^*^	**0.083**	0.000^*^	0.785	0.000^*^	0.000^*^	0.000^*^	0.291	0.191	0.993	**0.046**
$CN\to US$	0.097	0.361	0.396	0.750	0.667	0.853	0.069	0.062	0.007^*^	**0.027**	0.407
$US\to FR$	0.000^*^	**0.030**	0.003^*^	**0.036**	**0.021**	0.210	0.469	0.000^*^	0.000^*^	0.002^*^	0.232
$FR\to US$	0.798	0.637	0.134	0.148	**0.026**	0.322	0.151	**0.033**	0.179	0.001^*^	0.002^*^
$US\to GE$	0.000^*^	0.000^*^	0.002^*^	–	0.000^*^	0.299	0.000^*^	0.000^*^	0.955	0.000^*^	0.000^*^
$GE\to US$	0.950	0.146	0.843	–	0.148	0.471	0.251	0.778	**0.012**	**0.014**	0.118
$US\to JP$	0.804	0.000^*^	0.001^*^	0.648	0.676	**0.024**	0.632	0.479	0.000*	0.612	0.687
$JP\to US$	0.250	0.006^*^	0.451	0.941	0.882	0.058	0.784	0.000^*^	0.532	0.121	0.223
$US\to CH$	0.055	0.001^*^	0.000^*^	0.331	0.000^*^	–	**0.046**	0.000^*^	–	–	0.187
$CH\to US$	0.317	**0.039**	0.445	0.120	0.000^*^	–	0.598	0.001^*^	–	–	0.367
$US\to UK$	0.859	0.010^*^	0.009^*^	0.659	0.006^*^	**0.028**	0.000^*^	0.834	**0.027**	0.532	0.626
$UK\to US$	0.951	0.298	0.569	0.903	0.786	0.535	0.471	0.805	0.089	0.720	0.317

This table presents the p-values of the Granger causality in distribution of volatility. Data cover different periods, all ending on 17/05/2021 (see Table 1). The tests in the left tail are conducted considering $\alpha$ = 1%, 5% and 10%, while the tests in the right tail consider $\alpha$ = 90%, 95% and 99%. The tests are conducted pairwise between the US and other 6 countries (Canada (CN), France (FR), Germany (GE), Japan (JP), China (CH), and the UK) for 11 industries (Basic Materials (BM), Consumer Discretionary (CD), Consumer Staples (CS), Energy (EN), Financials (FI), Health Care (HC), Industrials (IN), Real Estate (RE), Technology (TEC), Telecommunications (TEL), and Utilities (UT)). $US\to k$ indicates Granger causality from the US to the returns of country $k$, and $k\to US$ indicates Granger causality from country $k$ to the US, for each industry. Numbers in bold indicate the rejection of the null of no Granger causality at the 5% level. One asterisk, “*”, denotes significance at the 1% level. “-” indicates that it was not possible to obtain reliable estimates of the Conditional Autoregressive Value-at-Risk (CAViaR) due to small sample size

The analyses of causality in volatility lead-lag during recession periods and the left tail of returns and right tail of volatility are related. According to Chen (2018), investors seek to hedge against market volatility because rising volatility does not incentivize investment opportunities. In other words, phases of high volatility tend to concur with drawdowns in stock markets, which may reduce investor confidence (Campbell and Hentschel 1992).

### Out-of-sample tests

In this subsection, we analyze our out-of-sample models using both statistical and economic performance measures. Table 13 displays the R-squared out-of-sample ${R}_{OOS}^{2}$ for return forecasts using the VAR model.

**Table 13:** R-squared out-of-sample (%)

	BM	CD	CS	EN	FI	HC	IN	RE	TEC	TEL	UT
$US\to CN$	− 1.02	− 0.86	− 0.90	− 0.84	− 0.93	− 0.13	− 0.44	− 1.04	− 0.7	− 0.41	0.30
$CN\to US$	− 0.38	− 0.29	0.21	− 1.32	− 0.33	− 0.31	− 0.30	− 0.22	0.44	− 0.90	0.52
$US\to FR$	**0.95**	− 0.89	− 0.18	− 0.98	0.10	− 0.14	− 1.15	− 0.77	− 0.18	− 0.15	0.29
$FR\to US$	− 0.95	− 0.59	− 0.12	− 1.33	− 0.19	− 0.12	− 0.45	− 1.86	− 0.22	− 0.37	− 0.48
$US\to GE$	0.66	**1.84**	− 0.02	− 0.95	− 1.23	− 0.32	− 0.24	− 0.32	− 0.46	0.03	− 0.05
$GE\to US$	− 0.68	− 0.23	0.02	− 1.78	− 0.35	− 0.10	− 0.45	− 0.32	0.17	− 0.20	0.00
$US\to JP$	**2.20**	**1.89**	**0.94**	**2.35**	0.71	**2.32**	**3.13**	**1.80**	**3.09**	**1.34**	− 0.05
$JP\to US$	− 0.54	− 0.23	− 0.58	− 1.25	0.05	− 0.77	− 0.36	− 0.57	− 0.12	0.09	0.26
$US\to CH$	0.25	− 0.10	0.01	0.45	0.05	0.19	0.02	− 0.27	− 0.95	− 0.04	− 0.05
$CH\to US$	− 0.05	− 0.16	− 0.73	− 1.60	− 0.92	− 0.07	− 0.21	− 0.32	− 0.71	− 0.27	− 0.07
$US\to UK$	− 0.14	0.13	0.29	− 0.33	− 0.03	0.34	− 0.49	− 0.79	− 0.28	0.27	− 0.22
$UK\to US$	− 1.34	− 0.64	0.56	− 1.15	− 0.19	− 0.09	− 0.67	− 1.91	− 0.10	− 0.18	− 0.06

This table presents the R-squared out-of-sample (Eq. 26) for the return forecasts of each country (Canada (CN), France (FR), Germany (GE), Japan (JP), China (CH), and the UK) and industry (Basic Materials (BM), Consumer Discretionary (CD), Consumer Staples (CS), Energy (EN), Financials (FI), Health Care (HC), Industrials (IN), Real Estate (RE), Technology (TEC), Telecommunications (TEL), and Utilities (UT)). $US\to k$ refers to the prediction of country $k$ returns using the US returns, and $k\to US$ refers to the prediction of US returns using the country $k$ returns. Numbers in bold indicate the rejection of the null of no predictive ability at the 5% level, according to the MSPE-adjusted statistic (Clark and West 2007)

Clearly, there is stronger evidence of the ability of US returns to predict returns in other countries than in the opposite direction. There is no significant ${R}_{OOS}^{2}$ for the prediction of US returns, but 11 out of 66 ${R}_{OOS}^{2}$ are statistically significant at the 5% level for other countries, most of which correspond to Japan (this exceptional behavior of Japan was already highlighted by Berben and Jansen 2005 and Ji et al. 2020). The average ${R}_{OOS}^{2}$ is negative for US returns (− 0.43%) and slightly positive for other countries (0.10%). These results reinforce the evidence from the prior subsections that predictability runs mostly from the US to the remaining countries.

Table 14 presents the annualized alphas for the strategy that, for each country, takes a long position in the industry with the highest predicted return and a short position in the industry with the lowest return forecast.

**Table 14:** Annualized alphas (%) for the long-short strategies by industry

	CN	FR	GER	JP	CH	UK
$US\to k$	0.46	1.77	6.53*	1.05	10.70*	0.64
$k\to US$	0.82	1.04	2.04*	**0.96**	4.48*	− 0.27

This table presents the annualized alphas corresponding to the strategy that, each week and for each country (Canada (CN), France (FR), Germany (GE), Japan (JP), China (CH), and the UK), takes a long position in the industry with the highest predicted return and a short position in the industry with the lowest predictive return. $US\to k$ refers to the prediction of country k returns using the US returns, and $k\to US$ refers to the prediction of US returns using the country k returns. Numbers in bold indicate the rejection of the null of no predictive ability at the 5% level. One asterisk, “*”, denotes significance at the 1% level

This long-short best-industry strategy always generates positive alphas when US returns are used to forecast other countries’ returns, two of which are statistically significant at the 1% level. The alphas in the opposite direction are generally lower, although three are significant at the 5% level. Curiously, the most successful trading strategies explore the returns’ relations between the US, Germany, and Japan in both directions.

The results of the strategy that, for each industry, takes a long position in the non-US country with the highest predicted return and a short position in the non-US country with the lowest predicted return are presented in Table 15.

**Table 15:** Annualized alphas (%) for the long-short strategies by country

	BM	CD	CS	EN	FI	HC	IN	RE	TEC	TEL	UT
$US\to k$	− 0.37	1.13	**0.77**	6.10*	2.33*	7.79*	0.42	8.17*	4.08*	3.14*	1.96*

This table presents the annualized alphas corresponding to the strategy that, each week and for each industry (Basic Materials (BM), Consumer Discretionary (CD), Consumer Staples (CS), Energy (EN), Financials (FI), Health Care (HC), Industrials (IN), Real Estate (RE), Technology (TEC), Telecommunications (TEL), and Utilities (UT)), takes a long position in the non-US country with the highest predicted return and a short position in the non-US country with the lowest predictive return. $US\to k$ refers to the prediction of country k returns using the US returns. Numbers in bold indicate the rejection of the null of no predictive ability at the 5% level. One asterisk, “*”, denotes significance at the 1% level

This active strategy almost always delivers positive alpha values. Namely, eight out of 11 annualized alphas are significantly positive at the 5% level, seven of which maintain their significance at the 1% level.

Table 16 shows the annualized CER for a CRRA investor with a coefficient of relative risk aversion equal to three,^5^ which decides the fraction of their wealth to invest in the industry index and risk-free assets based on return and volatility forecasts.

**Table 16:** Annualized certainty equivalent returns (%) for a CRRA investor with relative risk aversion coefficient equal to three

	BM	CD	CS	EN	FI	HC	IN	RE	TEC	TEL	UT
$US\to CN$	1.42	6.06	2.18	− 1.15	4.64	7.60	3.34	3.21	1.45	3.74	3.40
	*− 8.70*	*− 0.24*	*2.90*	*− 11.94*	*− 0.20*	*− 19.60*	*4.65*	*1.71*	*− 2.33*	*1.16*	*2.69*
$CN\to US$	5.05	5.52	4.46	6.01	5.78	5.456	6.27	1.93	5.54	3.92	2.49
	*0.20*	*6.95*	*5.43*	*− 7.42*	*− 2.11*	*7.164*	*4.33*	*4.93*	*9.23*	*0.03*	*2.39*
$US\to FR$	4.39	6.13	4.51	3.44	3.11	5.50	6.23	5.66	5.92	2.30	3.09
	*1.90*	*1.69*	*1.16*	*− 10.54*	*− 10.26*	*− 0.40*	*− 0.97*	*− 4.25*	*− 1.31*	*1.03*	*− 12.90*
$FR\to US$	3.74	6.13	4.42	5.95	4.45	5.55	6.18	4.09	6.43	2.56	2.53
	*0.20*	*6.95*	*5.43*	*− 7.42*	*− 2.11*	*7.16*	*4.33*	*4.93*	*9.23*	*0.03*	*2.39*
$US\to GE$	4.12	3.45	4.64	3.99	5.26	5.26	4.10	5.18	4.60	3.53	1.71
	*0.04*	*− 2.62*	*2.27*	*− 10.23*	*− 5.75*	*5.21*	*− 1.63*	*7.86*	*5.17*	*− 5.86*	*− 7.76*
$GE\to US$	3.77	5.89	4.44	− 4.04	5.86	5.52	5.98	1.68	5.15	3.42	3.11
	*0.20*	*6.95*	*5.43*	*− 7.42*	*− 2.11*	*7.16*	*4.33*	*4.93*	*9.23*	*0.03*	*2.39*
$US\to JP$	5.62	4.10	4.19	3.40	4.74	4.97	4.86	5.87	4.58	4.82	3.44
	*− 2.98*	*− 0.18*	*− 0.37*	*− 9.52*	*− 7.76*	*3.24*	*0.21*	*− 3.62*	*− 0.87*	*2.90*	*− 8.30*
$JP\to US$	3.33	4.99	4.40	5.76	4.95	5.38	6.31	4.20	5.69	3.65	3.84
	*0.20*	*6.95*	*5.43*	*− 7.42*	*− 2.11*	*7.16*	*4.33*	*4.93*	*9.23*	*0.03*	*2.39*
$US\to CH$	1.34	3.43	2.99	0.94	2.69	6.13	3.96	5.22	5.61	3.04	1.42
	*− 13.59*	*− 1.86*	*− 6.56*	*− 7.28*	*− 5.45*	*5.94*	*− 6.58*	*− 1.65*	*11.52*	*− 17.49*	*− 10.20*
$CH\to US$	1.68	4.23	3.08	3.43	4.11	5.42	4.28	2.05	4.86	4.17	2.14
	*0.20*	*6.95*	*5.43*	*− 7.42*	*− 2.11*	*7.16*	*4.33*	*4.93*	*9.23*	*0.03*	*2.39*
$US\to UK$	2.26	4.13	3.23	3.75	2.18	3.61	4.62	5.10	5.44	2.32	1.44
	*− 10.11*	*− 3.55*	*1.31*	*− 11.62*	*− 10.70*	*− 0.87*	*0.32*	*− 11.61*	*2.43*	*− 9.08*	*− 3.20*
$UK\to US$	4.15	5.49	4.37	5.54	6.06	5.72	6.20	4.29	5.65	1.44	1.68
	*0.20*	*6.95*	*5.43*	*− 7.42*	*− 2.11*	*7.16*	*4.33*	*4.93*	*9.23*	*0.03*	*2.39*

This table presents the annualized certainty equivalent returns for a CRRA investor with a relative risk aversion coefficient equal to 3, that, for each country (Canada (CN), France (FR), Germany (GE), Japan (JP), China (CH), and the UK) and industry (Basic Materials (BM), Consumer Discretionary (CD), Consumer Staples (CS), Energy (EN), Financials (FI), Health Care (HC), Industrials (IN), Real Estate (RE), Technology (TEC), Telecommunications (TEL), and Utilities (UT)), chooses the fraction of her wealth to invest in the industry portfolio and the risk-free asset based on the return and volatility predictions. $US\to k$ refers to the prediction of country $k$ returns using the US returns, and $k\to US$ refers to the prediction of US returns using the country $k$ returns. For each industry and pair of countries the first number is the CER of the active strategies and the second number, in italic, is the CER of the buy-and-hold of the industry in the second country of each pair

For non-US countries, this strategy generally delivers positive CERs. The overall average CERs for these countries is 3.91%, which exceeds both the CER from an investment in the risk-free asset (1.15%) and the average CER from a buy-and-hold strategy (-3.26%), where the investor buys the industry portfolio of the non-US country and holds it throughout the investment horizon. When non-US returns and volatilities are used to predict US returns and volatilities, the average of the CERs is also positive (4.36%), but its outperformance relative to the buy-and-hold strategy (average of the CERs equals 3.18%) is lower than when the forecasts are conducted in the opposite direction.

### Robustness checks

To assess the sensitivity of our results to different model specifications, significance levels, and data, we performed several robustness checks.

First, we address the problem of multiple testing. It is well known that when a researcher conducts multiple tests, traditional *p* values may offer a distorted picture of the strength of the statistical significance (Rapach et al. 2019). To overcome this problem, we use the adaptive procedure proposed by Benjamini and Hochberg (2000), which controls the number of rejections of the null hypothesis for the false discovery rate.

The adjustment procedure results in a higher number of rejections of the null hypothesis of the absence of Granger causality in the mean from the US to other countries (Table 17, first row) and a reduction in the number of significant tests in the opposite direction (Table 17, second row). Broadly speaking, there is some evidence of an increase in cross-industry predictability from the US to other countries (see Table 17, rows 3–13). Regarding volatility, the adjustment produces a slight increase in the number of rejections of the null hypothesis (see Table 17, rows 14 and 15). When we split the data into recessionary and expansionary periods, the evidence of return predictability weakens (Table 17, rows 16–19). Finally, for the causality in quantiles tests, there is an increase in the number of rejections of the null hypothesis of no predictability for the left tail of the return distributions (Table 17, row 20) and the right tail of the volatility distributions (Table 17, row 26) when the US returns are used to forecast other countries’ returns. In the opposite direction, the adjustment to the *p* values of the causality in the quantile test reveals a weakening of the predictability of US returns (Table 17, rows 21, 23, 25, and 27). Overall, the adaptive procedure reinforces evidence that predictability runs mostly from the US to other countries.

**Table 17:** Rejections of the null hypothesis based on unadjusted and adjusted p-values

		5%		1%	
		Unadjusted	Adjusted	Unadjusted	Adjusted
Table 3	$US\to k$	49	53	41	44
	$k\to US$	11	9	7	3
Table 5	BM	50	60	44	52
	CD	40	53	26	36
	CS	43	45	30	33
	EN	48	51	40	45
	FI	28	29	16	13
	HC	31	34	19	17
	IN	40	47	30	34
	RE	35	35	25	22
	TEC	32	32	20	17
	TEL	20	19	15	15
	UT	40	37	27	24
Table 6	$US\to k$	61	65	59	63
	$k\to US$	59	64	58	60
Table 8 (recession)	$US\to k$	42	35	25	17
	$k\to US$	12	3	6	0
Table 8 (expansion)	$US\to k$	26	24	18	14
	$k\to US$	10	5	5	2
Table 11 (left tail)	$US\to k$	32	36	23	24
	$k\to US$	22	15	15	14
Table 11 (right tail)	$US\to k$	11	6	7	5
	$k\to US$	10	4	4	0
Table 12 (left tail)	$US\to k$	14	7	7	6
	$k\to US$	13	7	4	1
Table 12 (right tail)	$US\to k$	37	38	29	29
	$k\to US$	13	7	7	2

This table presents the number of rejections of the null hypothesis based in the adjusted (Benjamini and Hochberg 2000) and unadjusted *p* values. Table 3: Coefficients of VAR(1) and significance of Granger causality in the mean. Table 5: Cross-industry coefficients of VAR(1) and significance of Granger causality in the mean from the US to other countries. Table 6: Coefficients of VAR(1) and significance of Granger causality in volatility. Table 8: Coefficients of VAR(1) and significance of Granger causality in the mean during expansions and recessions. Table 11: Granger causality in distribution of returns. Table 12: Granger causality in distribution of the volatility. The industries in Table 5 are Basic Materials (BM), Consumer Discretionary (CD), Consumer Staples (CS), Energy (EN), Financials (FI), Health Care (HC), Industrials (IN), Real Estate (RE), Technology (TEC), Telecommunications (TEL), and Utilities (UT). $US\to k$ refers to the prediction of country k returns using the US returns, and $k\to US$ refers to the prediction of US returns using the country $k$ returns. $US\to k$ refers to the prediction of country k using data from the US returns, and $k\to US$ refers to the prediction of US using data from country $k$

Next, we derive causality and feedback statistics from VAR(2). The causality results were not meaningfully different from those obtained with VAR(1), except that fewer countries showed a lower relationship with the US. The Geweke feedback measures remained almost the same until the last significant digit.

Since some series started later than others, we also analyzed the sensitivity of our results to the inclusion of only a complete series for each industry. For instance, in the case of basic materials (BM), we had complete data for all countries after 1993. Therefore, we excluded 20 years of data for some countries, which forced us to discard a significant part of the series that potentially contained relevant information for our study. The results show some differences in the Geweke feedback measures. For instance, the US reported a smaller percentage of unilateral feedback than before, while France, Germany, Canada, and the UK reported larger percentages. This is possibly due to their level of integration, which has increased in recent years. Naturally, there were no significant differences for countries that had little data availability before (e.g., China).

Finally, we repeated the analysis using daily and monthly data. There was increasingly pronounced causality for daily and weekly data between the US and other countries than for weekly data. These patterns also exist when considering data partitioning into expansion and recession periods, as illustrated in Table 18, which shows the coefficients of VAR(1) and the significance of Granger causality in the mean for the daily and monthly data.

**Table 18:** Coefficients of the VAR(1) and significance of Granger causality in the mean for daily and monthly data

	BM	CD	CS	EN	FI	HC	IN	RE	TEC	TEL	UT
*Panel A: daily data*											
$US \to CN$	0.157*	0.207*	0.164*	0.178*	0.128*	0.157*	0.138*	0.067*	0.087*	0.086*	0.153*
$CN \to US$	− 0.012	− 0.053*	− 0.067*	− 0.058*	− 0.094*	− 0.032*	**− 0.023**	− 0.087*	0.005	− 0.058*	− 0.049*
$US \to FR$	**0.239ª**	0.304*	0.267*	0.290*	0.266*	0.279*	0.263*	0.100*	0.299*	0.116*	0.136*
$FR \to US$	− 0.026*	− 0.020*	− 0.018*	**0.001**	− 0.027*	**− 0.016**	− 0.024*	− 0.038*	− 0.001	− 0.003	− 0.023*
$US \to GE$	0.221*	0.143*	0.184*	0.058*	0.219*	0.196*	0.250*	0.070*	0.179*	0.183*	0.139*
$GE \to US$	− 0.026*	0.012	− 0.026*	− 0.008	− 0.039*	− 0.043*	− 0.027*	− 0.015	0.000	− 0.012	− 0.021*
$US \to JP$	0.322*	0.371*	0.370*	0.328*	0.330*	0.290*	0.404*	0.199*	0.338*	0.257*	0.193*
$JP \to US$	− 0.009	− 0.023*	**− 0.013**	0.003	− 0.008	− 0.014	**− 0.018**	− 0.006	− 0.007	− 0.001	**− 0.015**
$US \to CH$	0.340*	0.226*	0.206*	0.224*	0.206*	0.095*	0.214*	0.093*	0.002	0.142*	0.224*
$CH \to US$	− 0.004	− 0.005	− 0.004	− 0.004	− 0.012	− 0.006	− 0.003	0.002	− 0.051*	**− 0.017**	− 0.016*
$US \to UK$	0.343*	0.271*	0.248*	0.309*	0.281*	0.289*	0.245*	0.151*	0.247*	0.200*	0.142*
$UK \to US$	− 0.021*	− 0.024*	− 0.026*	0.001	**− 0.018**	− 0.022*	− 0.016	− 0.037*	− 0.010	**− 0.014**	− 0.038*
*Panel B: monthly data*											
$US \to CN$	− 0.194	0.088	− 0.067	− 0.156	− 0.084	− 0.055	0.082	0.074	0.258	**0.281**	0.147
$CN \to US$	0.189	0.045	0.051	0.122	− 0.006	0.029	0.023	0.011	− 0.010	− 0.002	− 0.309*
$US \to FR$	− 0.157	− 0.129	− 0.070	− 0.105	0.071	0.016	− 0.161	− 0.038	0.034	0.051	− 0.027
$FR \to US$	0.059	0.001	0.018	0.062	− 0.031	− 0.020	0.109	0.079	− 0.017	0.005	− 0.010
$US \to GE$	0.112	− 0.059	0.040	− 0.355*	0.089	− 0.007	− 0.014	− 0.071	0.039	0.464*	0.073
$GE \to US$	− 0.167	− 0.037	0.008	0.003	− 0.031	0.068	0.094	0.011	0.018	− 0.045	− 0.089
$US \to JP$	0.098	0.024	0.064	0.072	0.118	− 0.025	0.049	0.011	0.208	0.319	0.197
$JP \to US$	− 0.037	− 0.181	− 0.112	− 0.029	− 0.011	− 0.068	− 0.062	− 0.002	− 0.045	0.061	− 0.010
$US \to CH$	− 0.130	− 0.254	− 0.458	0.194	0.024	− 0.335	− 0.099	− 0.018	0.045	0.134	− 0.020
$CH \to US$	0.110	− 0.006	− 0.050	0.041	− 0.024	− 0.039	− 0.055	0.031	− 0.202	− 0.127	− 0.096
$US \to UK$	− 0.181	− 0.148	− 0.114	0.146	0.130	− 0.045	− 0.052	**− 0.265**	0.222	**0.360**	− 0.036
$UK \to US$	− 0.009	0.036	0.066	0.051	0.010	0.072	0.048	0.139	0.039	**0.168**	− 0.104

This table presents the coefficients of a bivariate VAR(1) and the significance of the Granger causality tests resulting from a bivariate VAR(1) using daily and monthly returns. Data cover different periods, all ending on 17/05/2021 (see Table 1). The tests are conducted pairwise between the US and other 6 countries (Canada (CN), France (FR), Germany (GE), Japan (JP), China (CH), and the UK) for 11 industries (Basic Materials (BM), Consumer Discretionary (CD), Consumer Staples (CS), Energy (EN), Financials (FI), Health Care (HC), Industrials (IN), Real Estate (RE), Technology (TEC), Telecommunications (TEL), and Utilities (UT)). $US \to k$ indicates Granger causality from the returns of US to the returns of country $k$, and $k \to US$ indicates Granger causality from country $k$ to the US, for each industry. Numbers in bold indicate the rejection of the null hypothesis of no Granger causality at the 5% level. One asterisk, “*”, denotes significance at the 1% level

Causality is almost absent in monthly data, only in five out of 66 pairs $US \to k$ and two out of 66 pairs $k \to US$, where $k$ represents the other countries in the sample, are significant at the 5% level, respectively. There are only two cases of significance, namely $CN \to US$ for utilities (UT) at the 5% level, with a negative sign, and $UK \to US$ for telecommunications (TEL), which suggests that it takes less than a month for markets to adjust to international information. With daily data, all the pairs $US \to k$, except telecommunications (TEL) for China, are positive and significant at the 1% level. The coefficients of the VAR(1) equation for the other countries are mostly negative (57 out of 66 cases, 86%); 39 cases are significant at 5%. A total of 38 are negative and 30 are significant at the 1% level. The comparison between weekly (see Table 3) and daily data reveals that the number of significant coefficients for the equations $US \to k$ at the 5% and 1% levels increases from 49 to 57 and from 40 to 65, respectively. The number of significant coefficients for the equations $US \to k$ at the 5% and 1% levels increases from ten to 39 and from seven to 30, respectively. The increase in significant coefficients and the number of negative coefficients support the claim that, most likely, these coefficients are biased due to non-synchronous trading (Boudoukh et al. (1994a) or that the daily data are too noisy (Smith et al. 1993).

## Conclusion

This study examines the linear interdependencies between international industries, with a focus on the relationships between the US and other six countries (Canada, France, Germany, Japan, China, and the UK). This study differs from previous research, which mainly focused on international stock indexes or firm-level returns, ignoring international inter-industry information transmission.

Our results, based on the bivariate Granger causality test, show that the weekly returns of US industries have a strong and significant causal relationship with most of the countries under scrutiny. The only exception is China, possibly due to international trading constraints. We also find that the returns of non-US countries have limited predictive ability over the returns of US industries, implying that the causal relationship is mainly asymmetric. This asymmetry is also supported by feedback measures. Contemporaneous feedback was the major contributor to total feedback, with an average weight of 94%. These results imply that most markets are highly integrated, and that, on average, most information transmission occurs within one week. The causality in volatility is stronger and less asymmetric.

The US basic materials and energy industries have the strongest and most significant causality to industries in other countries. This finding is highly plausible because firms in other industries rely heavily on commodities and fuel. Additionally, returns of commodity- and material-producing industries situated earlier in the production chain are frequently strongly connected to the returns of industries positioned later in the chain.

During expansionary periods, the US dominates in other countries, but there is less causality than in recession periods. During recessions, there is high linear dependence between countries, but they react to a larger delay in information in other markets. This is possible due to the high level of uncertainty reported during recessions. To the best of our knowledge, this is the first study to directly document international evidence supporting asymmetric reactions to the US industry during recessions.

We also analyze Granger causality in the distribution for both industry returns and volatilities. Our results reveal that other countries do not incorporate US industry shocks in a timely manner. In particular, countries exhibit a delayed reaction to news from the US, especially in the left tail of the distribution of returns and the right tail of the distribution of volatilities.

Regarding the economic value of return forecasts based on US industry indices, we show that long-short strategies deliver mostly positive and significant alphas. Furthermore, a constant relative risk-averse investor who uses returns and volatilities’ predictions generated by our model obtains certainty equivalent returns in excess of the risk-free rate.

In sum, we may conclude that the US plays a leadership role in international markets, which can be justified by the fact that the US is the world’s largest economy in terms of GDP and an important trading partner for many countries. In addition, the US market has been analyzed and scrutinized by investors worldwide. This deep analyst coverage and investor attention increases the impact of US macroeconomic fundamentals on other international markets.

Our findings support the claim that evidence of the US and other international stock returns in-sample and out-of-sample predictability underscores the need for policymakers, investors, and academics alike to systematically observe the dynamics between the US and other countries, especially the of basic materials (BM), energy (EN), and financials (FI) industries and to pay careful attention in periods of recession. The importance of financials has already been highlighted by Asafo-Adjei et al. (2021), who advise continuous observation of the relationship between financial sector development and economic growth across time while considering adverse shocks from global economic policy uncertainty. In addition, as suggested by Aye et al. (2017), they should observe key market events (e.g., the US bear market of 2007–2009 and 2007–2008 subprime credit crisis periods), regulatory events (e.g., the 2008 short sale bans and the 2011 Uptick Rule, which restricts short selling), and technological events (e.g., the 2001 Credit Suisse Advanced Execution Services (AES) launch). Aye et al. (2017) and Berben and Jansen (2005) point out that policymakers should use leading US information to proactively design appropriate monetary policies in advance to avoid impending crises and stabilize the global financial system. For instance, capital controls could delay the reaction of a country’s stock market to news about the US stock markets.

An increase in market integration may negatively impact international diversification. Co-movement across the entire return distribution reduces the benefits of portfolio diversification, especially during periods of financial downturn when diversification is most needed (Beine et al. 2010).

Our study had several limitations. First, there is compelling evidence that local and international correlations between returns, volatility, and distributions of assets, industries, and markets are time-varying and that these interrelationships are subjected to external uncertainty shocks (Aye et al. 2017; Beine et al. 2010; Henkel et al. 2011; Caporale et al. 2016). Berben and Jansen (2005) highlighted that in the late 1990s, international correlations remained the same for Japan but doubled among Germany, the UK, and the US relative to the previous decade. In the 1990s and following years, there were many worldwide shocks, including the 1994 Mexican peso collapse, which affected many Latin American countries; the 1997 Thai crisis, which ignited the East Asian crisis; the 1998 Russian crisis, which affected Mexico and other Latin American countries; the 1999 Brazilian devaluation; the 2001 crises in Argentina and Turkey; the global financial crisis of 2008–2009 triggered by the US subprime crisis in August 2009 (Ji et al. 2020), and the COVID-19 pandemic (the World Health Organization’s (WHO) communique on the outbreak identifying it as a Public Health Emergency of International Concern (PHEIC) on January 30, 2020, and a pandemic on March 11, 2020). The probability of dependence relations between the US and other G7 stock markets entering a high dependence regime has increased in the twenty-first century, especially during and after the 2008 financial crisis (Ji et al. 2020). However, sudden shocks worldwide, such as the COVID-19 pandemic, have distorted most economic activities. For instance, in the case of China, Liu et al. (2022) consider that demand for energy and its influencing factors are related to the total scale of the economy and conclude that although manufacturing and consumption were affected, services were more vulnerable to the shocks from the COVID-19 pandemic. COVID-19 also disconnects the relationship between fundamentals and stock market activity (Fromentin 2022) and heightens the level of uncertainty, rendering effective asset allocation and portfolio diversification part of the optimum choice for investors (Asafo-Adjei et al. 2021). Whereas the 2008 global financial crisis started in the US and gradually spread to the rest of the world with a significant time delay, the COVID-19 crisis was especially abrupt. The pandemic instantaneously brought the global economy to a standstill by simultaneously disrupting demand and supply lines worldwide owing to widespread lockdowns (Fromentin 2022). Further, there is some evidence that it decreased market informational efficiency, especially in the US and the UK (Ozkan 2021; Junior et al. 2021). More importantly, using data from the Chinese stock market, Wang and Liu (2022) show that the shocks from the COVID-19 pandemic to the market was heterogeneous across industries, and the panic caused by the pandemic expanded volatility in daily returns. However, they note that the impulse of the shock from the pandemic tended to fade over time.

Second, our predictions might benefit from including information about investor sentiment using opinion pools (Zha et al. 2021) or ensembles of econometric and machine learning models (see, for instance, Sebastião and Godinho 2021).

Third, to provide a more realistic view of trading strategies, trading costs should be incorporated (Stübinger 2019 and Li et al. 2022 consider a proportional trading cost of 0.25%). The results also highlight the need to explicitly incorporate the US’s leading role in building international asset pricing models, as suggested by Aye et al. (2017) and Haque (2011).

Some possible extensions to this work could be the inclusion of exogenous variables in VAR models, such as proxies for specific industry characteristics (e.g., institutional holdings, market share, firm size, and trading volume).

Our findings differ from the growing literature on lead-lag relations as we provide a direct statistical and economic analysis of the causal link between US industry returns and non-US countries’ industry returns. We believe that our findings and developments provide a thorough view of the complex dynamics presented in markets and international industries. Our study is particularly relevant to policymakers, regulators, and asset allocation practitioners, especially at the international level.

## Data Availability

The data used in this research were obtained from the Thomson Reuters DataStream database.

## Abbreviations

US: United States
G7: Group of seven. Informal grouping of seven of the world's most advanced economies: Canada, France, Germany, Italy, Japan, the United Kingdom, and the United States
UK: United Kingdom
GDP: Gross Domestic Product
BRIC: Brazil, Russia, India and China
VIX: Chicago Board Options Exchange Volatility Index
EU: European Union
STC-GARCH: Smooth-Transition Correlation Generalized AutoRegressive Conditional Heteroskedasticity
CoVaR: Conditional Value-at-Risk
BRICS: Brazil, Russia, India, China and South Africa
ICB: Industry Classification Benchmark
BM: Basic Materials industry
CD: Consumer discretionary industry
CS: Consumer staples industry
EN: Energy industry
FI: Financials industry
HC: Health care industry
IN: Industrials industry
RE: Real estate industry
TEC: Technology industry
TEL: Telecommunications industry
UT: Utilities industry
MSCI ACWI: Morgan Stanley Capital International All Country World Index
NBER: National Bureau of Economic Research
VAR(1): Vector autoregressive model of order one
AIC: Akaike information criterion
BIC: Schwartz information criterion
VaR: Value-at-risk
CAViaR: Conditional autoregressive value-at-risk
MSPE: Mean squared prediction error
CRRA: Constant relative risk aversion
CER: Certainty equivalent return
CN: Canada
FR: France
GE: Germany
JP: Japan
CH: China
WHO: World Health Organization’s
PHEIC: Public Health Emergency of International Concern

## Footnotes

1 Series that were in the domestic currency were converted to US dollars using the series of exchange rates, also obtained from the Thomson Reuters DataStream database.

2 As suggested in Candelon and Tokpavi (2016), we have tested the sensitivity of the results using $M = ln\left( T \right)$, $M = 1.5T^{0.3}$, and $M = 2T^{0.3}$, and different kernels functions (Bartlett, Daniell, and Parzen). Results remained broadly unchanged, hence we used $M = ln\left( T \right)$ and the Barlett kernel as these are the specifications usually used in the literature.

3 The main difference between the MSCI ACWI index and the MSCI World index is that the later focus on developed markets.

4 We assume that short-selling and borrowing are not allowed.

5 We also computed the CER for a CRRA investor with a coefficient of relative risk aversion equal to five. The conclusions remain broadly unchanged.

## References

[CR1] Arshanapalli B, Doukas J (1993). International stock market linkages: evidence from the pre-and post-October 1987 period. J Bank Finance.

[CR2] Asafo-Adjei E, Boateng E, Isshaq Z, Idun AAA, Owusu Junior P, Adam AM (2021). Financial sector and economic growth amid external uncertainty shocks: insights into emerging economies. PLoS ONE.

[CR3] Aye GC, Balcilar M, Gupta R (2017). International stock return predictability: is the role of US time-varying?. Empirica.

[CR4] Badrinath SG, Kale JR, Noe TH (1995). Of shepherds, sheep, and the cross-autocorrelations in equity returns. Rev Financ Stud.

[CR5] Beine M, Cosma A, Vermeulen R (2010). The dark side of global integration: increasing tail dependence. J Bank Finance.

[CR6] Benjamini Y, Hochberg Y (2000). On the adaptive control of the false discovery rate in multiple testing with independent statistics. J Educ Behav Stat.

[CR7] Berben RP, Jansen WJ (2005). Comovement in international equity markets: a sectoral view. J Int Money Finance.

[CR8] Bollerslev T, Osterrieder D, Sizova N, Tauchen G (2013). Risk and return: Long-run relations, fractional cointegration, and return predictability. J Financ Econ.

[CR9] Bollerslev T, Marrone J, Xu L, Zhou H (2014). Stock return predictability and variance risk premia: statistical inference and international evidence. J Financ Quant Anal.

[CR10] Boudoukh J, Richardson M, Whitelaw R (1994). A tale of three schools: insights of autocorrelations of short-horizon stock returns. Rev Financ Stud.

[CR11] Boudoukh J, Richardson M, Whitelaw R (1994). Industry’s returns and the Fisher effect. J Finance.

[CR12] Brennan MJ, Jegadeesh N, Swaminathan B (1993). Investment analysis and the adjustment of stock prices to common information. Rev Financ Stud.

[CR13] Buncic D, Gisler KI (2016). Global equity market volatility spillovers: a broader role for the United States. Int J Forecast.

[CR14] Cambón MI, Vaduva MA (2017). Lead-lag patterns in the Spanish and other European equity markets. Span Rev Financ Econ.

[CR15] Camilleri SJ, Scicluna N, Bai Y (2019). Do stock markets lead or lag macroeconomic variables? Evidence from select European countries. N Am J Econ Finance.

[CR16] Campbell JY, Hentschel L (1992). No news is good news: an asymmetric model of changing volatility in stock returns. J Financ Econ.

[CR17] Candelon B, Tokpavi S (2016). A nonparametric test for Granger causality in distribution with application to financial contagion. J Bus Econ Stat.

[CR18] Caporale GM, Gil-Alana LA, Orlando JC (2016). Linkages between the US and European stock markets: a fractional cointegration approach. Int J Finance Econ.

[CR19] Chen W (2018). Lost in internationalization: rise of the renminbi, macroprudential policy, and global impacts. J Int Econ Law.

[CR20] Chordia T, Swaminathan B (2000). Trading volume and cross-autocorrelations in stock returns. J Finance.

[CR21] Clark T, West K (2007). Approximately normal tests for equal predictive accuracy in nested models. J Econom.

[CR22] Conrad J, Kaul G (1988). Time variation in expected returns. J Bus.

[CR23] Copeland M, Copeland T (1998). Leads, lags, and trading in global markets. Financ Anal J.

[CR24] Dutta A (2018). Implied volatility linkages between the US and emerging equity markets: a note. Glob Finance J.

[CR25] Engle RF, Manganelli S (2004). CAViaR: conditional autoregressive value at risk by regression quantiles. J Bus Econ Stat.

[CR26] Fan N, Fan ZP, Li Y, Li M (2022). Does the lead-lag effect exist in stock markets?. Appl Econ Lett.

[CR27] Fromentin V (2022). Time-varying causality between stock prices and macroeconomic fundamentals: connection or disconnection?. Finance Res Lett.

[CR28] Geweke J (1982). Measurement of linear dependence and feedback between multiple time series. J Am Stat Assoc.

[CR29] Granger CW (1969). Investigating causal relations by econometric models and cross-spectral methods. Econometrica.

[CR30] Griffin JM, Hirschey NH, Kelly PJ (2011). How important is the financial media in global markets?. Rev Financ Stud.

[CR31] Haque T (2011). Lead–lag effects in Australian industry portfolios. Asia-Pac Financ Mark.

[CR32] Henkel SJ, Martin JS, Nardari F (2011). Time-varying short-horizon predictability. J Financ Econ.

[CR33] Hong H, Torous W, Valkanov R (2007). Do industries lead stock markets?. J Financ Econ.

[CR34] Hong Y, Liu Y, Yang S (2009). Granger causality in risk and detection of extreme risk spillover between financial markets. J Econom.

[CR35] Hou K (2007). Industry information diffusion and the lead-lag effect in stock returns. Rev Financ Stud.

[CR36] Jacobsen B, Marshall BR, Visaltanachoti N (2019). Stock market predictability and industrial metal returns. Manag Sci.

[CR37] Jegadeesh N, Titman S (1995). Overreaction, delayed reaction, and contrarian profits. Rev Financ Stud.

[CR38] Jeong K, Härdle WK, Song S (2012). A consistent nonparametric test for causality in quantile. Econom Theory.

[CR39] Ji Q, Liu BY, Cunado J, Gupta R (2020). Risk spillover between the US and the remaining G7 stock markets using time-varying copulas with Markov switching: evidence from over a century of data. N Am J Econ Finance.

[CR40] Jiang Z, Yoon SM (2020). Dynamic co-movement between oil and stock markets in oil-importing and oil-exporting countries: two types of wavelet analysis. Energy Econ.

[CR41] Junior PO, Adam AM, Asafo-Adjei E, Boateng E, Hamidu Z, Awotwe E (2021). Time-frequency domain analysis of investor fear and expectations in stock markets of BRIC economies. Heliyon.

[CR42] Kanas A, Kouretas GP (2005). A cointegration approach to the lead–lag effect among size-sorted equity portfolios. Int Rev Econ Finance.

[CR43] Khalfaoui R, Tiwari AK, Kablan S, Hammoudeh S (2021). Interdependence and lead-lag relationships between the oil price and metal markets: fresh insights from the wavelet and quantile coherency approaches. Energy Econ.

[CR44] Kou G, Akdeniz ÖO, Dinçer H, Yüksel S (2021). Fintech investments in European banks: a hybrid IT2 fuzzy multidimensional decision-making approach. Financ Innov.

[CR45] Kou G, Yüksel S, Dinçer H (2022). Inventive problem-solving map of innovative carbon emission strategies for solar energy-based transportation investment projects. Appl Energy.

[CR46] Lee CM, Sun ST, Wang R, Zhang R (2019). Technological links and predictable returns. J Financ Econ.

[CR47] Li Y, Wang T, Sun B, Liu C (2022). Detecting the lead–lag effect in stock markets: definition, patterns, and investment strategies. Financ Innov.

[CR48] Liu X, An H, Li H, Chen Z, Feng S, Wen S (2017). Features of spillover networks in international financial markets: evidence from the G20 countries. Physica A.

[CR49] Liu L, Huang J, Li H (2022). Estimating the real shock to the economy from COVID-19: the example of electricity use in China. Technol Econ Dev Econ.

[CR50] Lo AW, MacKinlay AC (1990). An econometric analysis of nonsynchronous trading. J Econom.

[CR51] Lo AW, MacKinlay AC (1990). When are contrarian profits due to stock market overreaction?. Rev Financ Stud.

[CR52] Menzly L, Ozbas O (2010). Market segmentation and cross-predictability of returns. J Finance.

[CR53] Moskowitz TJ, Grinblatt M (1999). Do industries explain momentum?. J Finance.

[CR54] Nyberg H, Pönkä H (2016). International sign predictability of stock returns: the role of the United States. Econ Model.

[CR55] Ozkan O (2021). Impact of COVID-19 on stock market efficiency: evidence from developed countries. Res Int Bus Finance.

[CR56] Parsons CA, Sabbatucci R, Titman S (2020). Geographic lead-lag effects. Rev Financ Stud.

[CR57] Rapach DE, Strauss JK, Zhou G (2013). International stock return predictability: what is the role of the United States?. J Finance.

[CR58] Rapach DE, Strauss JK, Tu J, Zhou G (2019). Industry return predictability: a machine learning approach. J Financ Data Sci.

[CR59] Roll R (1992). Industrial structure and the comparative behavior of international stock market indices. J Finance.

[CR61] Salisu AA, Gupta R, Pierdzioch C (2022). Predictability of tail risks of Canada and the US over a century: the role of spillovers and oil tail risks. N Am J Econ Finance.

[CR62] Sarwar G, Khan W (2017). The effect of US stock market uncertainty on emerging market returns. Emerg Mark Finance Trade.

[CR63] Sebastião H, Godinho P (2021). Forecasting and trading cryptocurrencies with machine learning under changing market conditions. Financ Innov.

[CR64] Shiller RJ (2000). Measuring bubble expectations and investor confidence. J Psychol Financ Mark.

[CR65] Smith KL, Brocato J, Rogers JE (1993). Regularities in the data between major equity markets: evidence from Granger causality tests. Appl Financ Econ.

[CR66] Stübinger J (2019). Statistical arbitrage with optimal causal paths on high-frequency data of the S&P 500. Quant Finance.

[CR67] Troster V, Penalva J, Taamouti A, Wied D (2021). Cointegration, information transmission, and the lead-lag effect between industry portfolios and the stock market. J Forecast.

[CR68] Tse Y (2018). Return predictability and contrarian profits of international index futures. J Futures Mark.

[CR69] Wang Q, Liu L (2022). Pandemic or Panic? A firm-level study on the psychological and industrial impacts of COVID-19 on the Chinese stock market. Financ Innov.

[CR70] Wen YC, Lin PT, Li B, Roca E (2015). Stock return predictability in South Africa: the role of major developed markets. Finance Res Lett.

[CR71] Zeng K, Mills EFEA (2021). Can economic links explain lead–lag relations across firms?. Int J Finance Econ.

[CR72] Zha Q, Kou G, Zhang H, Liang H, Chen X, Li CC, Dong Y (2021). Opinion dynamics in finance and business: a literature review and research opportunities. Financ Innov.

[CR73] Ahnert T, Bertsch C (2022) A wake-up call theory of contagion. ECB working paper no. 2022/2658. https://ssrn.com/abstract=4100188

[CR74] Baumöhl E, Lyócsa Š (2012) Constructing weekly returns based on daily stock market data: a puzzle for empirical research? MPRA Paper 43431. https://mpra.ub.uni-muenchen.de/43431/

[CR75] Croce MM, Marchuk T, Schlag C (2019) The leading premium (No. w25633). National Bureau of Economic Research, working paper no. 2563. https://www.nber.org/system/files/working_papers/w25633/w25633.pdf

[CR76] Forbes KJ (2012) The 'Big C': identifying and mitigating contagion. MIT Sloan Research Paper No. 4970–12. https://ssrn.com/abstract=2149908

[CR77] Rapach DE, Strauss J, Tu J, Zhou G (2015) Industry interdependencies and cross-industry return predictability*.* Research Collection Lee Kong Chian School of Business. https://ink.library.smu.edu.sg/lkcsb_research/4515

[CR78] Rizova S (2010) Predictable trade flows and returns of trade-linked countries. AFA 2011 Denver meetings paper. https://ssrn.com/abstract=1562697

[CR79] Venditti F, Veronese G (2020) Global financial markets and oil price shocks in real time*. *European Central Bank (ECB), working paper series no. 2472. https://www.ecb.europa.eu/pub/pdf/scpwps/ecb.wp2472~611f104931.en.pdf

[CR80] World Bank (2021) World Bank Open Data Website. http://data.worldbank.org. Accessed 16 May 2021

